# Aging differentially alters the transcriptome and landscape of chromatin accessibility in the male and female mouse hippocampus

**DOI:** 10.3389/fnmol.2024.1334862

**Published:** 2024-01-22

**Authors:** Jennifer M. Achiro, Yang Tao, Fuying Gao, Chia-Ho Lin, Marika Watanabe, Sylvia Neumann, Giovanni Coppola, Douglas L. Black, Kelsey C. Martin

**Affiliations:** ^1^Department of Biological Chemistry, David Geffen School of Medicine, UCLA, Los Angeles, CA, United States; ^2^Department of Psychiatry and Biobehavioral Sciences, Semel Institute for Neuroscience and Human Behavior, David Geffen School of Medicine, UCLA, Los Angeles, CA, United States; ^3^Department of Microbiology, Immunology and Molecular Genetics, UCLA, Los Angeles, CA, United States

**Keywords:** aging, sex bias, hippocampus, gene expression, alternative splicing, ATAC-seq, chromatin accessibility, LINE-1

## Abstract

Aging-related memory impairment and pathological memory disorders such as Alzheimer’s disease differ between males and females, and yet little is known about how aging-related changes in the transcriptome and chromatin environment differ between sexes in the hippocampus. To investigate this question, we compared the chromatin accessibility landscape and gene expression/alternative splicing pattern of young adult and aged mouse hippocampus in both males and females using ATAC-seq and RNA-seq. We detected significant aging-dependent changes in the expression of genes involved in immune response and synaptic function and aging-dependent changes in the alternative splicing of myelin sheath genes. We found significant sex-bias in the expression and alternative splicing of hundreds of genes, including aging-dependent female-biased expression of myelin sheath genes and aging-dependent male-biased expression of genes involved in synaptic function. Aging was associated with increased chromatin accessibility in both male and female hippocampus, especially in repetitive elements, and with an increase in LINE-1 transcription. We detected significant sex-bias in chromatin accessibility in both autosomes and the X chromosome, with male-biased accessibility enriched at promoters and CpG-rich regions. Sex differences in gene expression and chromatin accessibility were amplified with aging, findings that may shed light on sex differences in aging-related and pathological memory loss.

## Introduction

Aging is associated with cognitive decline and memory impairment, including spatial and episodic memory deficits (Nyberg et al., 2012). Research has revealed sex differences in memory performance with aging, as well as sex differences in the incidence of Alzheimer’s disease, a condition associated with memory loss and damage to the hippocampal brain region (Gao et al., 1998; Herlitz and Rehnman, 2008; Jack et al., 2015; McCarrey et al., 2016; Beam et al., 2018; Anstey et al., 2021; Davis et al., 2021). The hippocampus is critical for spatial, episodic, and long-term memory formation (Bird and Burgess, 2008), and therefore, understanding how sex differences impact aging in the hippocampus can provide insights into aging- and sex-dependent declines in memory.

Numerous studies have shown that the abundance of cell types in the brain remains largely constant during aging (Stilling et al., 2014; Ximerakis et al., 2019; Hajdarovic et al., 2022; Allen et al., 2023; Hahn et al., 2023); however, increased immune activation, blood–brain barrier breakdown, altered synaptic function, as well as changes in gene expression, alternative splicing, epigenetic marks, and chromatin accessibility have been associated with aging in the brain (Shankar et al., 1998; Morrison and Baxter, 2012; Stilling et al., 2014; Barrientos et al., 2015; Barter and Foster, 2018; Ximerakis et al., 2019; Zhang et al., 2022; Bieri et al., 2023). Regulated chromatin accessibility is critical to the precise control of gene expression patterns (Su et al., 2017), and hippocampus-dependent long-term memory formation requires new gene expression (Bambah-Mukku et al., 2014; Eagle et al., 2016; Marco et al., 2020). However, few studies have examined sex differences in aging-related changes in gene expression in the hippocampus (Mangold et al., 2017; Hadad et al., 2019), and none, to the best of our knowledge, have examined how sex differences affect aging-related alternative splicing and chromatin accessibility in the hippocampus. Understanding how gene regulatory networks and chromatin dynamics associated with aging differ between sexes in the hippocampus may guide sex-dependent therapies for age-related memory disorders.

## Materials and methods

### Animals

Male and female C57BL6/J mice, aged 8 weeks and 78 weeks, were purchased from Jackson Laboratories. The use of mice in this study was approved by the UCLA Institutional Animal Care and Use Committee. Mice were housed in groups under a 12:12 h light/dark cycle with food provided *ad libitum* until they reached the age of 10 weeks (young adult) and 80 weeks (aged). For sample collection, mice were deeply anesthetized with isoflurane and euthanized by cervical dislocation. The brain was quickly removed and cooled for 1 min in ice-cold PBS before the hippocampus from each hemisphere was dissected on ice and processed immediately. For sequencing experiments, one hippocampus was homogenized in Trizol for RNA preparation, and the other hippocampus was homogenized for nuclei preparation for ATAC-seq (see below). For qPCR and Western blot validation, hippocampal tissue was homogenized in Trizol or RIPA buffer, respectively (see below).

### RNA-seq library preparation and sequencing

RNA was extracted using a Trizol/RNeasy hybrid protocol. In brief, after phase separation, the aqueous phase was mixed with one volume of 70% ethanol and passed through an RNeasy spin column (QIAGEN RNeasy Mini Kit). RNA quality was determined using the high-sensitivity RNA ScreenTape assay (Agilent 5067-5579), and all samples had RIN scores >7.0. RNA-seq libraries for differential expression or splicing analysis were prepared for four biological replicates/conditions, including four young adult males, four aged males, four young adult females, and four aged females per library type. For differential expression analysis, total RNA libraries were prepared using the TruSeq Stranded Total RNA library prep kit with Ribo-Zero (Illumina 20020596). Paired-end 100 bp sequencing to a depth of ~50 million reads per sample was performed using the Illumina HiSeq 2500 system at the UCLA Broad Stem Cell Research Center Sequencing Core. For splicing analysis, mRNA libraries were prepared with poly-A selection using the TruSeq Stranded mRNA library prep kit (Illumina 20020594), and paired-end 75 bp sequencing to a depth of ~50 million reads per sample was performed using the Illumina HiSeq 2500 system at the UCLA Neuroscience Genomics Core.

### ATAC-seq library preparation and sequencing

ATAC-seq sample preparation was performed for eight biological replicates/conditions according to detailed protocols obtained from Hongjun Song’s lab at the University of Pennsylvania (Buenrostro et al., 2015; Su et al., 2017). In brief, one hippocampus was homogenized with a Dounce tissue grinder (Wheaton 357544) in 2 mL HB buffer (1 mM DTT, 0.15 mM spermine, 0.5 mM spermidine, protease inhibitor (Sigma-Aldrich 04693159001), 0.3% IGEPAL-630, 0.25 M sucrose, 25 mM MgCl_2_, 20 mM Tricine-KOH). The homogenate was filtered through a 40-μm strainer and centrifuged over one volume of cushion buffer (0.5 mM MgCl_2_, 0.5 mM DTT, protease inhibitor, 0.88 M sucrose) at 2,800*g* for 10 min in a swinging bucket centrifuge at 4°C. The nuclei pellet was resuspended in 20 μL PBS. Then, 1 μL of the nuclei sample was stained with Hoechst 33342 to calculate nuclei concentration, and 50,000 nuclei per sample were used for downstream library preparation. Libraries were prepared using the Nextera DNA library prep kit (Illumina FC-121-1030), and quality was analyzed using the D1000 ScreenTape Assay (Agilent 5067-5582). Paired-end 50 bp sequencing to a depth of ~40 million reads per sample was performed using the Illumina HiSeq 2500 system at the UCLA Broad Stem Cell Research Center Sequencing Core.

### RNA-seq data analysis

RNA-seq reads were mapped to the mouse genome (mm10) using STAR with the two-pass option (Dobin et al., 2013). Only uniquely mapped reads were used for downstream analysis. Lowly expressed genes were removed by retaining only genes with counts per million >0.1 in at least four samples (17,821 genes). Read counts were normalized via the trimmed mean method before differential expression analysis (DEA) (Robinson and Oshlack, 2010). For DEA, the analysis package edgeR (Robinson et al., 2010) was used, with an FDR <0.05 cutoff. For aging-dependent expression differences, data from female and male samples were analyzed separately. For differential splicing analysis, the analysis package rMATS version 4.0.2 (Shen et al., 2014) was used. Alternative splicing events were included for analysis if events had FDR-adjusted *p*-value <0.05, total reads ≥50, and skipping/junction reads >20. For gene ontology enrichment analysis, the Database for Annotation, Visualization, and Integrated Discovery (DAVID) online tool was used (Huang et al., 2009; Huang da et al., 2009). RNA-sequencing data have been deposited in NCBI’s Gene Expression Omnibus (Edgar et al., 2002) and are accessible through GEO Series accession number GSE244506.^1^

### ATAC-seq data analysis

Eight replicates per condition were sequenced for ATAC-seq; however, one young male sample had less than one million reads and was not used in further analyses. Adapter content was trimmed using Scythe,^2^ and ATAC-seq reads were mapped to the mouse genome (mm10) using Bowtie 2 (Langmead and Salzberg, 2012). Reads in blacklist regions (Dunham et al., 2012; Amemiya et al., 2019) and reads mapped to mitochondria were removed. Peaks were called on each individual sample using MACS3 (with parameter settings—nomodel-f BAMPE and FDR cutoff of 0.05) (Zhang et al., 2008). For each sample, read pairs (fragments) were counted for each peak using featureCount (Liao et al., 2014) (with parameter settings—p—countReadPairs–M—fraction). For quality control, we calculated a transcription start site (TSS) enrichment score using ATACseqQC (Ou et al., 2018) and a FRiP score (fraction of reads/fragments in peaks). One young male and one aged male sample had a TSS enrichment score < 10 and/or a FRiP score < 0.2; therefore, these samples were excluded from further analyses. The remaining samples had an average TSS enrichment score of 20.2 ± 1.1 (mean ± s.e.m.) and an average FRiP score of 0.46 ± 0.03 (mean ± s.e.m.). For each condition, narrow peak sets were merged, and peaks were included in consensus peak sets for each condition if there was at least a 50% overlap with a peak in at least two replicates (consensus sets: 65,088 peaks in young male, 71,719 in aged male, 61,264 in young female, and 68,655 in aged female). A total consensus peak set (92,233 peaks) was generated by merging the consensus peak sets from each condition. Fragments were counted for each total consensus peak in each sample using featureCount as described above, and differential analysis was performed using DESeq2, including sequencing batch as a variable (Love et al., 2014). Batch correction for principal components analysis was performed using limma (Ritchie et al., 2015). Peak annotation, peak histograms, and motif searches were done using HOMER (Heinz et al., 2010). Browser track examples were generated from the UCSC genome browser^3^ (Kent et al., 2002). For the ATAC-seq peak profile heat map, normalized peak histograms for each peak were generated in HOMER for each sample using 10 bp bins. Then, for each peak, an average ATAC histogram was calculated for each condition and ranked by young male ATAC signal intensity ± 100 bp around the peak center. For ATAC-seq profile plots, normalized peak histograms for each sample were generated in HOMER using 10 bp bins, producing one histogram per sample based on the number of peaks (or gene TSSs) indicated in figures, and then averages and standard error of the means were calculated based on the sample histograms (with *n* = number of samples, i.e., 6 young male, 7 aged male, 8 young female, and 8 aged female). Genomic locations of full-length intact long interspersed element-1 (LINE-1) regions were obtained using L1Base 2 (Penzkofer et al., 2017). Where indicated in the results, we used smooth-quantile with GC-content normalization (QSmooth-GC) using qsmooth (Hicks et al., 2017; Van den Berge et al., 2022). This study used computational and storage services associated with the Hoffman2 Shared Cluster provided by UCLA Institute for Digital Research and Education’s Research Technology Group. ATAC-sequencing data have been deposited in NCBI’s Gene Expression Omnibus (Edgar et al., 2002) and are accessible through GEO Series accession number GSE244506.^4^

### qPCR

Samples for qPCR validation were prepared from separate animals from those used for library preparation. Then, 50 ng of total RNA was reverse transcribed into cDNA using SuperScript III First-Strand Synthesis System (Invitrogen 18080051) with random hexamer primers for four biological replicates of each sex and age group. Technical triplicates were prepared for each primer set using SYBR Green PCR Master Mix (Applied Biosystems 4309155). The following primers were used:

*Gapdh (F: AGGTCGGTGTGAACGGATTTG; R: TGTAGACCATGTAGTTGAGGTCA),

*Tubb3 (F: TAGACCCCAGCGGCAACTAT; R: GTTCCAGGTTCCAAGTCCACC),

*Gfap (F: CCCTGGCTCGTGTGGATTT; R: GACCGATACCACTCCTCTGTC),

*Pcdhb9 (F: ACTGCTCTTGAGAATACCAGAGA; R: AGGACGTGAAAATAAGGGTTGG),

*Gpr17* (F: TCACAGCTTACCTGCTTCCC; R: CCGTTCATCTTGTGGCTCTTG),

*Ptpro (F: AACATCCTGCCGTATGACTTTAG; R: GGGACTTCTGTTGTAGGACCATC),

*Npnt (F: GGACAGGTCCGATGTCAGTG; R: CTTCCAGTCGCACATTCATCA),

*Mag (F: CTGCCGCTGTTTTGGATAATGA; R: CATCGGGGAAGTCGAAACGG),

*Mbp (F: GACCATCCAAGAAGACCCCAC; R: GCCATAATGGGTAGTTCTCGTGT),

LINE-1 5’UTR (F: TGAGTGGAACACAACTTCTGC; R: CAGGCAAGCTCTCTTCTTGC),

LINE-1 ORF1 (F: ATGGCGAAAGGCAAACGTAAG; R: ATTTTCGGTTGTGTTGGGGTG),

LINE-1 5’UTR:ORF1 (F: CTGCCTTGCAAGAAGAGAGC; R: AGTGCTGCGTTCTGATGATG).

Gene primers from PrimerBank^5^ are indicated with a *. Primers for LINE-1 were obtained from previous publications (De Cecco et al., 2013; Van Meter et al., 2014). The rest of the gene primers were custom-designed to span exon-exon junctions. The samples were run on the Bio-Rad CFX Connect real-time PCR detection system. We used the delta–delta Ct method for calculating fold gene expression, with delta Cts calculated relative to *Gapdh* (for RNA-seq validation) or *Tubb3* (for LINE-1) and delta–delta Cts calculated relative to young male samples. Results were tested for normality using the Shapiro–Wilk test. Unpaired *t*-tests/one-way ANOVAs with Sidak’s multiple comparisons test or Mann–Whitney/Kruskal–Wallis tests with Dunn’s multiple comparison tests were used for normally and non-normally distributed data, respectively.

### Western blot

Western blot samples were prepared from four biological replicates. For each replicate, one hippocampus was homogenized in a RIPA buffer with protease and phosphatase inhibitors and centrifuged at 10,000*g* for 10 min at 4°C to remove cell debris. Protein concentration was determined using the Pierce BCA protein assay kit (Thermo Fisher 23225). All protein samples were diluted to a protein concentration of 0.5 mg/mL. Then, 4X sample loading buffer (0.2 M Tris–HCl pH 6.5, 4.3 M glycerol, 8.0% (w/v) SDS, 6 mM bromophenol blue, and 0.4 M DTT) was added, and the samples were boiled at 95–100°C for 10 min. The samples were then aliquoted and stored at −20°C. Samples were loaded on NuPAGE 4–12% Bis-Tris protein gels (Invitrogen NP0335BOX) and transferred onto 0.2 μm nitrocellulose membranes. After transfer, blots were blocked with Odyssey TBS blocking buffer (LI-COR 927-50000) for 1 h at room temperature before being incubated in the primary antibody at 4°C overnight. The following primary antibodies were used: anti-MBP (Abcam, ab218011) and anti-TUJ1 (Biolegend, 801201). Blots were incubated in secondary antibodies for 2 h at room temperature. Blots were imaged using the LI-COR Odyssey imaging system and quantified using LI-COR Image Studio software.

## Results

**Figure 1:**
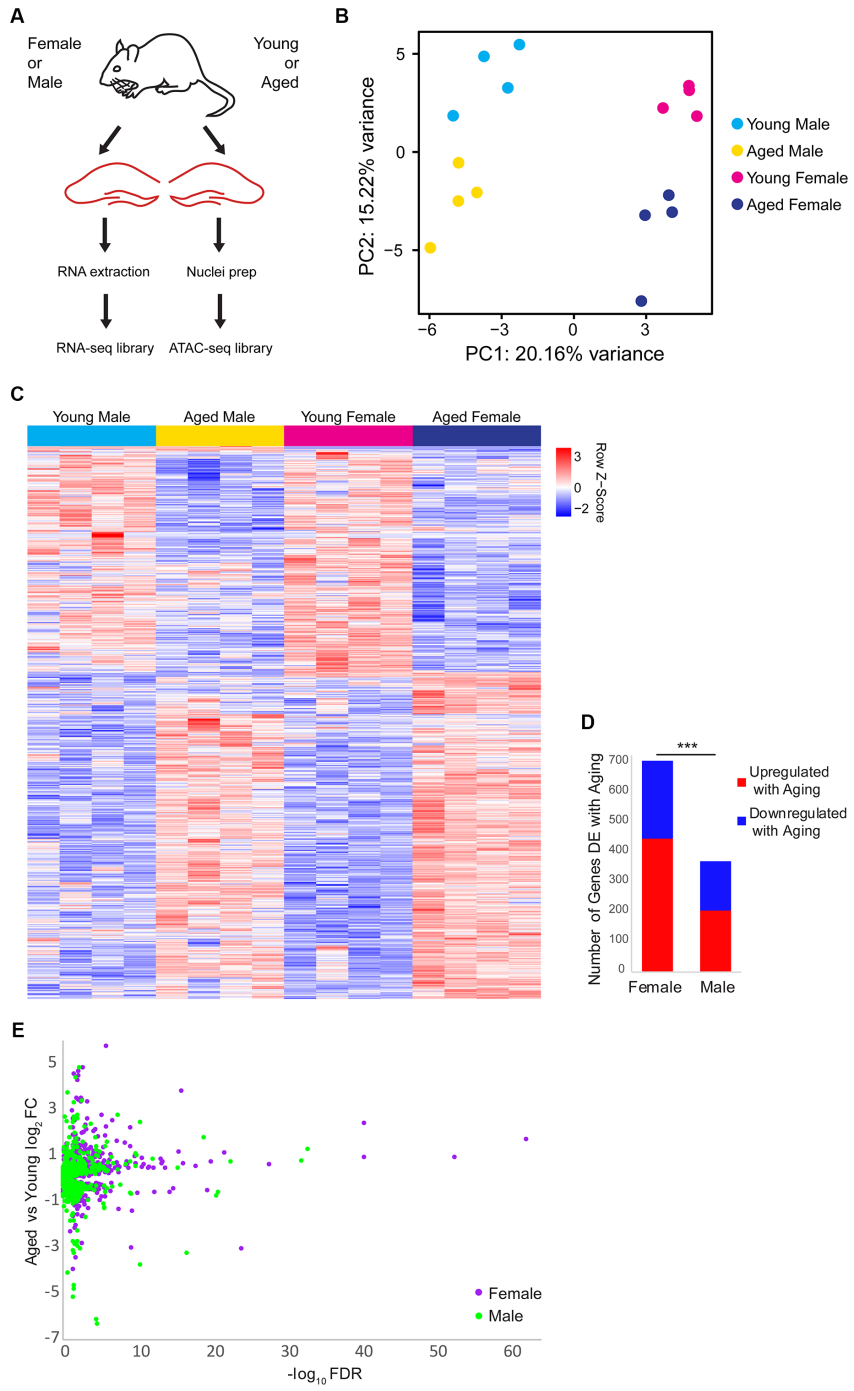
Gene expression changes with aging and sex bias in female and male mouse hippocampus. **(A)** Illustration of the experimental design. **(B)** Principle component analysis (PCA) of RNA-seq gene expression data. *N* = four biological replicates for each group (young adult female, aged female, young adult male, and aged male). **(C)** Expression heat map of 905 sex-biased or aging-related differentially expressed (DE) genes (FDR < 0.05). Each row represents a gene and each column is a biological replicate. Red row *z*-scores indicate high relative expression and blue indicates low relative expression (see Supplementary Table S1 for all FPKM and log_2_FC values). **(D)** Number of genes that are DE between aged and young animals in females and males (FDR < 0.05). *** indicates *p* < 0.001 for the chi-square test. **(E)** Volcano plot of aging-related DE genes in males (green) and females (purple).

To investigate sex differences in aging-related changes in the transcriptome (including alternative splicing) and chromatin accessibility in the hippocampus, we performed RNA-sequencing (RNA-seq) and Assay for Transposase-Accessible Chromatin sequencing (ATAC-seq) (Buenrostro et al., 2013, 2015) on the hippocampus of young adult (10 weeks) or aged (80 weeks) mice of both sexes (Figure 1A). Principle component analysis of the RNA-seq data showed that samples were well-separated by sex and age by the first and second principal components, respectively (Figure 1B). Overall, 905 genes were differentially expressed (DE) either by sex or aging, with the largest number of differences occurring with aging irrespective of sex (Figure 1C; Supplementary Table S1). In the hippocampus of females, we found 446 genes that were upregulated and 261 genes downregulated with aging. Significantly fewer aging-associated changes were detected in the hippocampus of males compared to females (chi-square test, *p* < 0.001, *X^2^* = 108.1), with 206 and 164 genes being upregulated and downregulated in males, respectively, with aging (Figures 1D,E). These results indicate that aging is associated with widespread changes in gene expression, with a larger number of genes undergoing aging-related changes in expression in the hippocampus of females than males.

### Aging results in changes in the expression of genes involved in cell adhesion, immune function, and neuronal development in both sexes

**Figure 2:**
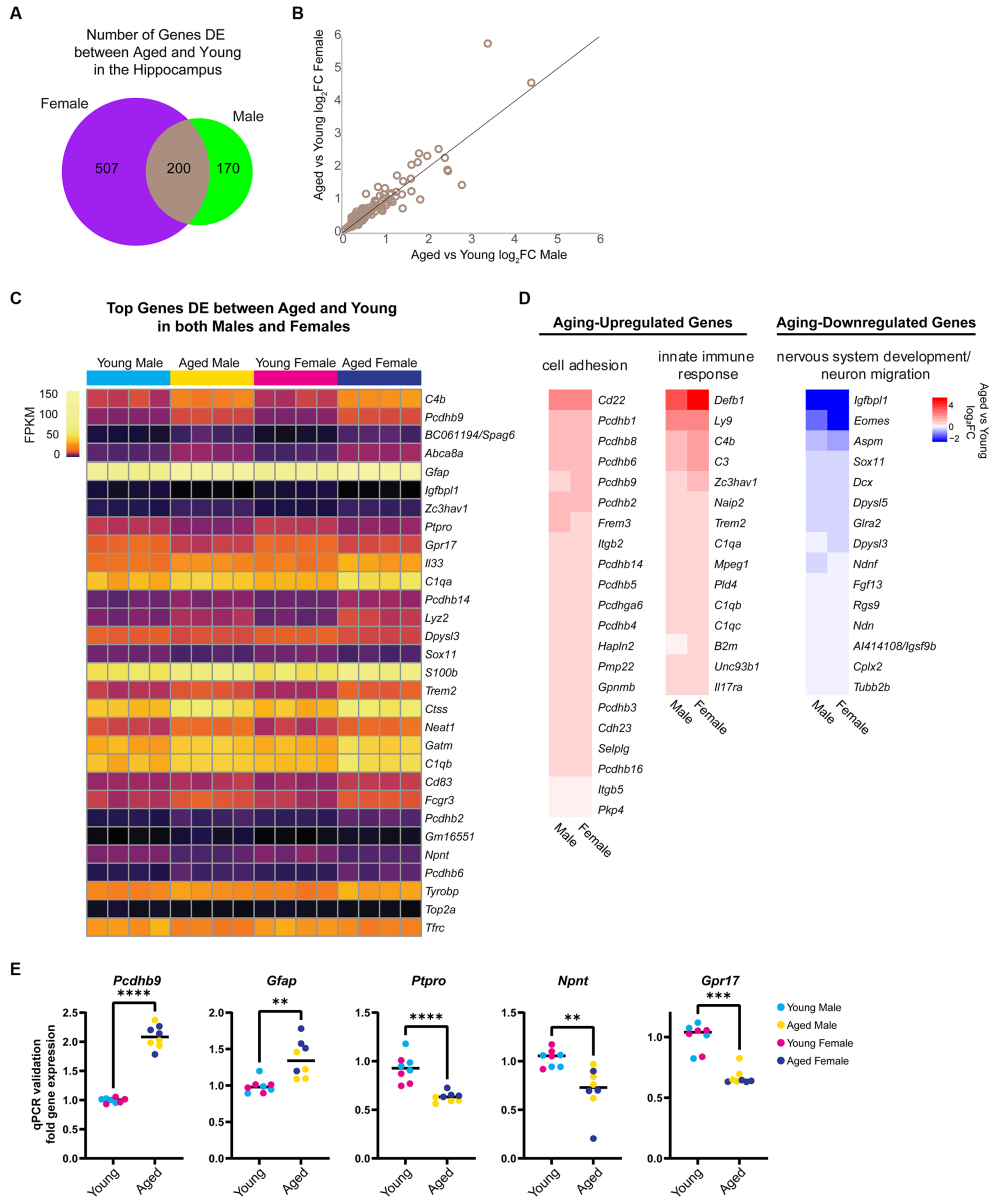
Aging is associated with changes in the expression of cell adhesion, immune response, and nervous system development genes. **(A)** Venn diagram of number of genes DE (FDR < 0.05) with aging in female and male mouse hippocampus. **(B)** Scatter plot of aged vs. young adult log_2_FC in males and females for the 200 genes that showed aging-related differential expression in the hippocampus of both sexes. **(C)** Expression heat map of top 30 genes (sorted by lowest FDR) DE in both male and female hippocampus with aging. Each row represents a gene and each column is a biological replicate. FPKM for each gene/replicate is represented by color (see Supplementary Table S1 for all FPKM and log_2_FC values). **(D)** Fold change heat maps of genes in biological process categories from gene ontology analysis that were upregulated (red) and downregulated (blue) in the hippocampus with aging. **(E)** qPCR validation for genes *Pcdhb9*, *Gfap*, *Ptpro*, *Npnt*, and *Gpr17* in young and aged male and female hippocampus (*n* = four each sex/age). *Pcdhb9* unpaired *t*-test *p* < 0.0001; *Gfap* unpaired *t*-test *p* = 0.001; *Ptpro* unpaired *t*-test *p* < 0.0001; *Npnt* unpaired *t*-test *p* = 0.003; *Gpr17* Mann–Whitney test *p* < 0.001. ** indicates *p* < 0.01, *** indicates *p* < 0.001, **** indicates *p* < 0.0001.

First, we focused on sex-independent gene expression changes with aging. We found that 136 genes were upregulated and 64 genes were downregulated with aging in both sexes (Figure 2A). We noted that although these genes were DE with aging in both sexes, for many genes, the aging-related fold change in female hippocampus was larger than in male hippocampus (Figure 2B). We ranked DE genes by lowest FDR in either males or females and found that many of the aging-dependent genes encoded immune response genes, protocadherins, extracellular matrix proteins, and neurodevelopment-related proteins (Figure 2C). Gene ontology analysis revealed that genes upregulated with aging showed enrichment for cell adhesion (GO:0007155, FDR = 5.92E-08) and innate immune response (GO:0045087, FDR = 0.02), whereas genes downregulated with aging showed enrichment for nervous system development (GO:0007399, FDR = 5.38E-04) and neuron migration (GO:0001764, FDR = 0.002; Figure 2D). To validate these findings, we performed qPCR using a separate cohort of animals for a number of aging-dependent genes (Figure 2E). We also analyzed gene expression changes from an existing dataset in a mouse model of Alzheimer’s disease (AD) (Forner et al., 2021). This analysis showed that, similar to our finding with aging, more genes showed AD-related changes in the female hippocampus compared to the male AD hippocampus (Supplementary Figure S1A). Furthermore, we found that ~30% of the aging-related genes we identified also showed AD-related differential expression in the hippocampus, including immune response genes that showed AD-related changes in both young adult and aged AD mice (Supplementary Figure S1B). These results are consistent with previous reports showing similar aging-related changes in the brain, including increases in immune-response genes and decreases in neurodevelopment-related genes (Walter et al., 2011; Uddin and Singh, 2013; Stilling et al., 2014; Barrientos et al., 2015; Carlock et al., 2017; Lanke et al., 2018; Ximerakis et al., 2019; Li M. et al., 2020; Rivera et al., 2021; Hahn et al., 2023).

### More genes show sex-biased expression in the hippocampus with aging, including higher expression of myelin sheath-related genes in aged females compared to aged males

**Figure 3:**
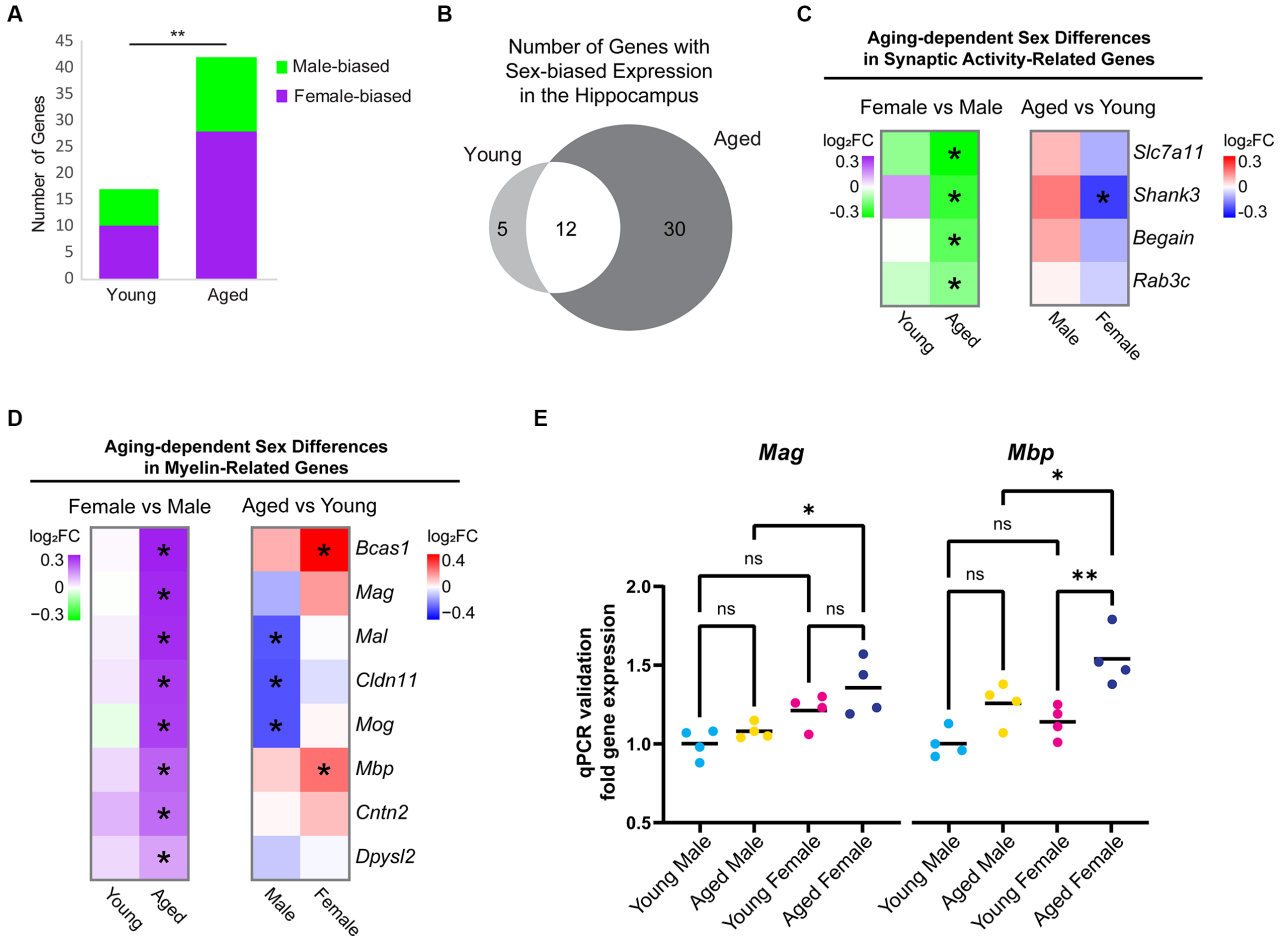
Sex differences in expression of hippocampal genes during aging. **(A)** Number of genes that were DE between female and male hippocampus (FDR < 0.05) in young adult and aged animals. Purple indicates higher expression in female and green indicates higher expression in male. ** indicates *p* < 0.01 by chi-square test. **(B)** Venn diagram of sex-biased DE genes in the hippocampus. **(C)** Fold change heat maps of synaptic activity-related genes showing higher expression in aged male vs. female hippocampus. Left heat map shows sex-biased differential expression (purple indicates higher in female and green indicates higher in male). Right heat map shows aging-related differential expression (red indicates upregulation with aging and blue indicates downregulation with aging).* indicates significant differential expression (FDR < 0.05). **(D)** Fold change heat maps [as in **(C)**] of myelin-related genes showing sex-biased expression. **(E)** qPCR validation for myelin sheath genes *Mag* and *Mbp* in young adult and aged male and female hippocampus (*n* = four for each condition). *Mag* aged male vs. aged female ANOVA with Sidak’s multiple comparison test adjusted *p* = 0.022. *Mbp* aged male vs. aged female ANOVA with Sidak’s multiple comparison test adjusted *p* = 0.039; young female vs. aged female adjusted *p* = 0.004. *indicates *p* < 0.05, ** indicates *p* < 0.01, ns = not significant.

To determine whether and how sex impacts aging-dependent changes in gene expression, we compared gene expression profiles between the hippocampus of young adult males and females and between aged males and females. In the hippocampus of young animals, we identified 17 sex-biased genes. Among these, neuropeptide Y (*Npy)*, a neuropeptide whose expression is correlated with female-biased stress-related memory disorders (Nahvi and Sabban, 2020), showed a 1.3-fold higher expression in the hippocampus of young females compared to young males, but a female-specific downregulation with aging (young female vs. male log_2_FC = 0.41, FDR < 0.001; average FPKM: young male = 64.34, aged male = 67.38, young female = 88.11, aged female = 71.34). In the hippocampus of aged animals, significantly more genes (42) were DE between sexes (chi-square test, *p* = 0.002, *X^2^* = 9.8; Figure 3A). Most of the sex-biased genes in the hippocampus of young animals also showed sex bias in the hippocampus of aged animals (Figure 3B). Of the genes with sex-biased expression in both young and aged, all were on sex chromosomes except for *Prl* (prolactin) and *Cplx2* (complexin 2). Complexin 2, a protein involved in synaptic vesicle fusion and whose dysregulated expression is associated with a number of cognitive disorders (Brose, 2008; Hass et al., 2015), was more highly expressed in males compared to females in both age groups, and this gene was also significantly downregulated with aging in both sexes (female vs. male: young log_2_FC = −0.16, aged log_2_FC = −0.21; aged vs. young: male log2FC = −0.16, female = −0.21). Of the sex-biased genes located on the X chromosome, all are known to escape X-inactivation in the mouse brain (Deng et al., 2014). Interestingly, we also found that *Xist*, a female-specific non-coding RNA critical for X-inactivation (Pandya-Jones and Plath, 2016; Galupa and Heard, 2018), was significantly upregulated in the female hippocampus with aging (log_2_FC = 0.21, FDR = 0.009; average FPKM: young male = 0.06, aged male = 0.04, young female = 27.75, aged female = 32.08). This finding is consistent with a recent report showing increased *Xist* expression with aging in the mouse hypothalamus (Hajdarovic et al., 2022). Altogether, these results suggest that aging results in more divergence in the hippocampal transcriptome between males and females.

To further explore sex differences in gene expression that arise with aging, we focused on the 30 genes with sex-biased expression only in aged animals. Eight genes were male-biased (*Slc7a11/xCT, Begain*, *Rab3c*, *Shank3*, *Hist1h4d, D830046C22Rik*, *Ncl*, and *Scarf2*), the first four of which encode proteins involved in synaptic transmission (Figure 3C) (Deguchi et al., 1998; Schlüter et al., 2004; Kouser et al., 2013; Massie et al., 2015). We found that 22 genes showed female-biased expression in aged hippocampus, including the myelination-related gene, *Bcas1*, which showed an aging-related increase in expression in female but not male hippocampus, resulting in significantly higher expression in aged females than aged males (log_2_FC = 0.36, FDR < 0.001; average FPKM: young male = 33.4; aged male = 37.3, young female = 33.8, aged female = 47.9).

Gene ontology analysis of female-biased genes in aged hippocampus revealed a significant enrichment of the term myelin sheath (GO:0043209; FDR < 0.001). We then assessed all genes associated with the GO terms “myelin sheath” and “myelination” (GO:0043209 and GO:0042552) and found that 8 genes (*Bcas1*, *Ma*g, *Mal*, *Mbp*, *Cldn11*, *Cntn2*, *Mog*, and *Dpysl2*) had sex-biased expression in the hippocampus of aged animals, all of which showed a female bias (Figure 3D). In contrast, other oligodendrocyte-related genes such as *Olig1*, *Olig2*, *Opalin*, *Cnp*, *Plp1, Myrf, Gpr17*, and *Mobp* were not DE between aged female and aged male hippocampus, suggesting that this was not due to a difference in the number of oligodendrocytes or oligodendrocyte precursor cells (OPCs), as has been previously reported (Hahn et al., 2023). Furthermore, we found no sex bias in the expression of myelination-related genes (GO:0043209 and GO:0042552) in young adult animals. The sex bias in myelin genes in aged hippocampus resulted from aging-related changes in both males and females: aging resulted in a significant downregulation of *Mog*, *Cldn11*, and *Mal* in the hippocampus of males but not females, whereas aging resulted in significant upregulation in *Bcas1* and *Mbp* in the hippocampus of females but not males (Figure 3D). We also performed qPCR for two of the myelin sheath genes, *Mag* and *Mbp*, using samples from a separate cohort of animals and verified that the expression of both had female-biased expression in aged hippocampus (Figure 3E). This sex-biased expression of myelin sheath genes suggests that the hippocampus of aged females may have less myelin degeneration or more remyelination than aged males.

### Alternative splicing of myelin sheath-related genes changes with aging in both sexes

**Figure 4:**
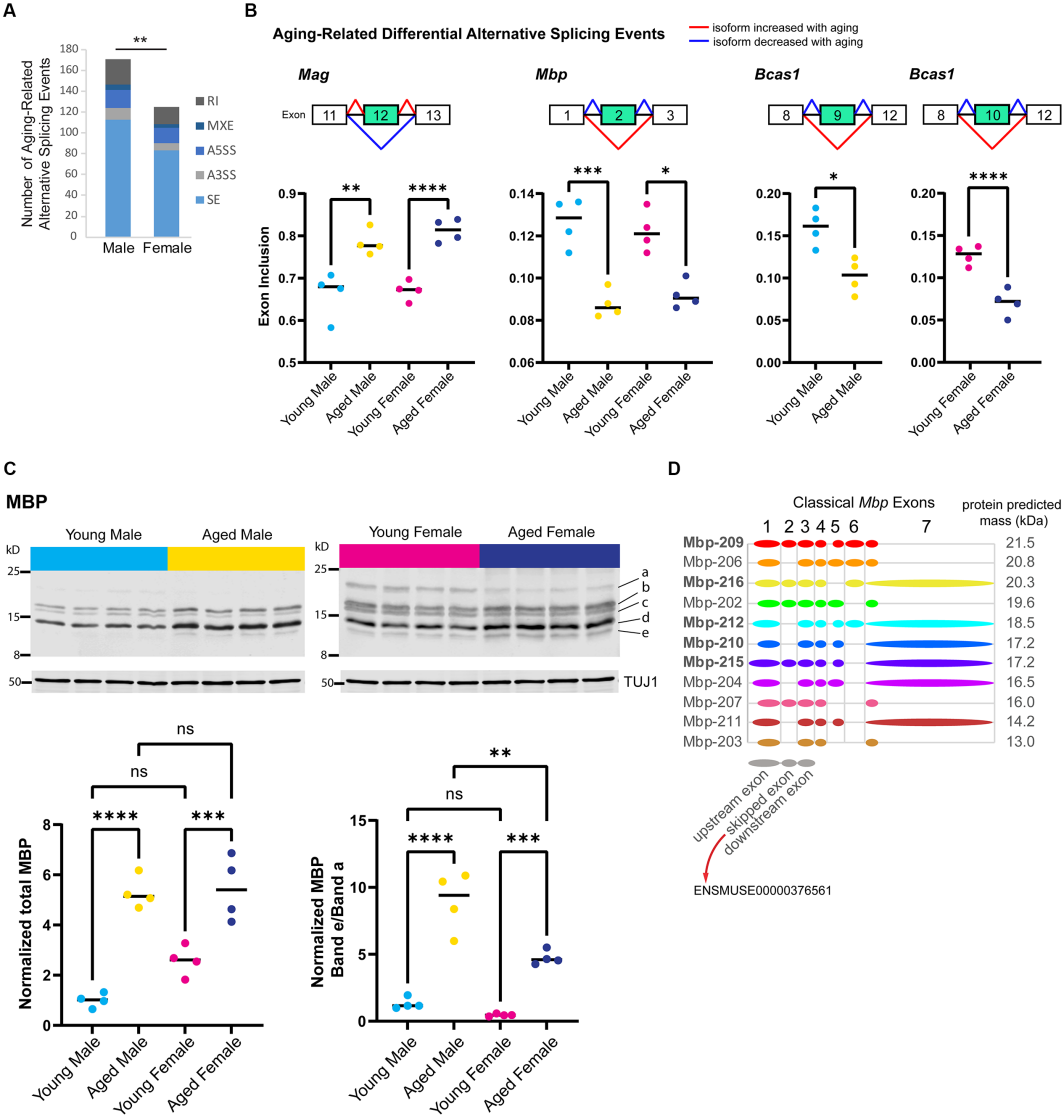
Aging is associated with alternative splicing of myelin sheath-related genes. **(A)** Number of each type of aging-related AS event in male and female hippocampus. SE = skipped exon, A5SS = alternative 5′ splice site, A3SS = alternative 3’splice site, MXE = mutually exclusive exons, RI = retained intron. **(B)** Genes that exhibited aging-related alternative splicing events in both male and female hippocampus. Under each gene name is a diagram showing the skipped and flanking exons involved in each alternative splicing event. Below the diagram, plots show exon inclusion values and statistical significance calculated from rMATS (Shen et al., 2014). *Mag* aging-related inclusion of exon 12 (ENSMUSE00000373997) in males rMATS FDR = 0.001 and females FDR < 0.0001. *Mbp* aging-related skipping of exon 2 (ENSMUSE00000376561) in males FDR < 0.001 and females FDR = 0.016. *Bcas1* aging-related skipping of exons 9–11 (ENSMUSE00000170506, ENSMUSE00000170504, ENSMUSE00001234083) in males FDR = 0.029 and female FDR < 0.0001 (also see Supplementary Figures S2A–C). **(C)** Western blot analysis for MBP protein isoforms in young adult and aged hippocampus. Top row shows Western blot images for MBP protein and loading control TUJ1 (below) for young adult and aged male and female hippocampus (4 biological replicates each). Letters on the right side indicate the bands that were quantified. Bottom panels show the quantification of total MBP (left plot) and the ratio of the lowest-migrating MBP isoform (e) to the highest-migrating MBP isoform **(a)**. See Supplementary Figures S2D,E for quantification of each band. Total MBP young male vs. aged male ANOVA with Sidak’s multiple comparison test adjusted *p* < 0.0001; young female vs. aged female adjusted *p* = 0.001. MBP band e/a young male vs. aged male ANOVA with Sidak’s multiple comparison test adjusted *p* < 0.0001; young female vs. aged female adjusted *p* = 0.001; aged male vs. aged female adjusted *p* = 0.001. **(D)** Classical (not including Golli) *Mbp* protein-coding splice variants from Ensembl (Cunningham et al., 2021) release 102 with a diagram of included exons. Diagrams of upstream, skipped, and downstream exons are shown in gray below splice variant diagrams. Bolded transcript names indicate Ensembl’s designation as stable, reviewed, and high-quality transcript annotations. The predicted protein mass from Ensembl for the product of each transcript is indicated to the right of the diagram. ** indicates *p* < 0.01, *** indicates *p* < 0.001, **** indicates *p* < 0.0001, ns = not significant.

Alternative splicing patterns have been reported to be altered with aging in the mouse hippocampus and show sex-specific differences in the human brain (Trabzuni et al., 2013; Stilling et al., 2014; Bhadra et al., 2020). To examine sex bias and aging-related changes in alternative splicing, we generated a poly-A selected RNA-library from the hippocampus of young adult and aged male and female mice and queried five types of alternative splicing (AS) events: skipped exon (SE), alternative 5′ splice site (A5SS), alternative 3’splice site (A3SS), mutually exclusive exons (MXE), and retained introns (RI). We identified 591 significant AS events in 452 genes that showed sex bias or aging-related changes (FDR < 0.05, Supplementary Table S2). Aging in the male hippocampus resulted in 171 AS events in 154 genes, significantly more events than that occurred with aging in the female hippocampus (Figure 4A; 125 AS events in 124 genes; chi-square test, *X^2^* = 7.2, *p* = 0.007).

First, we focused on sex-independent AS in the hippocampus with aging and identified seven genes (*Mag, Bcas1, Mbp, 4732471J01Rik, Huwe1, Ctxn3, and Mthfsl)* with skipped exon events that showed the same aging-related AS event in both males and females. The top three most significant aging-related AS events occurred in myelin-related genes: (1) inclusion of exon 12 (ENSMUSE00000373997) in *Mag*, (2) skipping of exon 2 (ENSMUSE00000376561) in *Mbp*, and (3) skipping of exons 9–11 (ENSMUSE00000170506, ENSMUSE00000170504, ENSMUSE00001234083) in *Bcas1* (Figure 4B; Supplementary Figures S2A–C). As mentioned previously, the overall transcript abundance of myelin sheath genes *Mag*, *Mbp*, and *Basc1* were female-biased in the aged hippocampus (Figure 3D); however, the proportions of exon inclusion for these genes were similar between the sexes. The aging-related skipping of exon 2 in *Mbp* was also supported at the protein level: aged animals of both sexes exhibited an overall increase in MBP protein compared with young animals but specifically showed a loss of a higher-migrating MBP species and a gain of a low-migrating species that correspond to *Mbp* splice variants with the inclusion or skipping of exon 2, respectively (Figures 4C,D; Supplementary Figures S2D,E). These aging-related changes in *Mag* and *Mbp* exon skipping are similar to those that have been reported to occur throughout development and aging (Tropak et al., 1988; Fujita et al., 1998; Woodruff and Franklin, 1998; Li et al., 2000; Sugiyama et al., 2002). In addition to *Mag* and *Mbp*, *Bcas1* is also known to be involved in myelination (Darbelli et al., 2017; Ishimoto et al., 2017); therefore, aging results in changes in the expression of different isoforms of myelin-related genes.

### Sex-biased alternative splicing in the young and aged hippocampus

**Figure 5:**
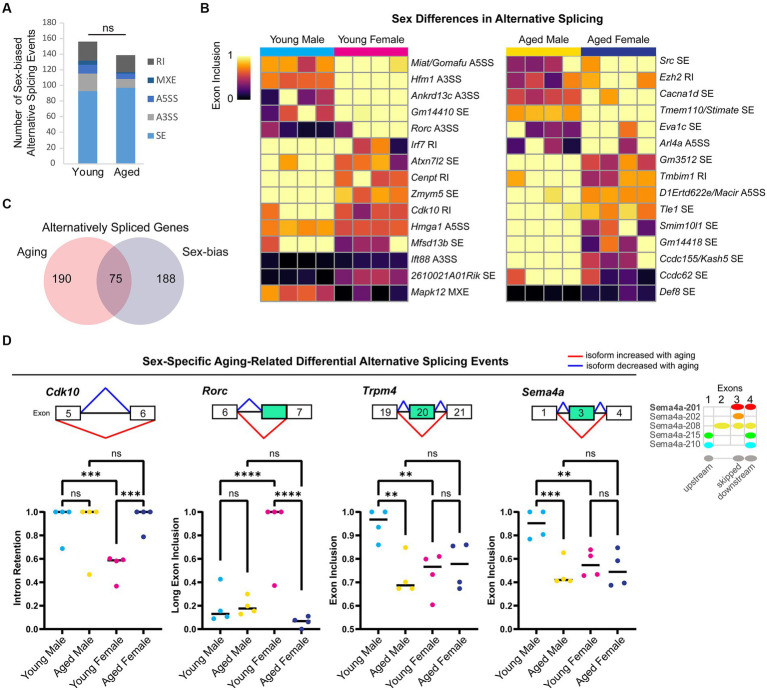
Sex differences in alternative splicing in the hippocampus. **(A)** Number of each type of sex-biased AS event in young adult and aged hippocampus. SE = skipped exon, A5SS = alternative 5′ splice site, A3SS = alternative 3’splice site, MXE = mutually exclusive exons, RI = retained intron. ns = not significant. **(B)** Exon inclusion heat map of top most significant sex-biased AS events in young adult or aged hippocampus (by rMATS FDR). Each column represents a biological replicate, and each row represents an AS event labeled on the right. **(C)** Venn diagram showing the number of genes with aging-related or sex-biased AS events. **(D)** Example genes with sex-specific aging-related alternative splicing events in the hippocampus. Under each gene name is a diagram showing the skipped and flanking exons involved in each alternative splicing event. Below the diagram, plots show exon inclusion values and statistical significance calculated from rMATS. *Cdk10* female bias in exclusion of intron between exons 5 (ENSMUSE00000292260) and 6 (ENSMUSE00000292228) in young hippocampus (FDR < 0.001) and aging-related intron retention in females (FDR < 0.001). *Rorc* female bias in long exon inclusion of exon 7 (ENSMUSE00000566508) in young hippocampus (FDR < 0.0001) and aging-related short exon usage in females (FDR < 0.0001). *Trpm4* male bias in exon inclusion of exon 20 (ENSMUSE00000594904) in young hippocampus (FDR < 0.01) and aging-related exon skipping in males (FDR < 0.01). *Sema4a* male bias in exon inclusion of exon 3 (ENSMUSE00000832345) in the young hippocampus (FDR < 0.01) and aging-related exon skipping in males (FDR < 0.001). Diagram on the right shows Ensembl protein-coding transcripts for *Sema4a* with the skipped exon corresponding to the second exon of Sema4a-208. ** indicates *p* < 0.01, *** indicates *p* < 0.001, **** indicates *p* < 0.0001, ns = not significant.

To determine whether and how sex impacts AS, we compared AS profiles of male and female young adult and aged hippocampus. We found 156 sex-biased AS events in 144 genes in young adult hippocampus and 139 sex-biased AS events in 129 genes in aged hippocampus (Figure 5A), including 9 genes with sex-biased AS in both young and aged (*Ankrd13c, Ccdc62, Epn2, Lrrc14, Lrrcc1, Mapk12, Pofut2, Rtel1*, and *Tle1*). We ranked the sex-biased AS events by lowest FDR-adjusted *p*-value in either the young or aged hippocampus and found that within the 30 most significant autosomal AS events (Figure 5B), many occurred in genes encoding nuclear proteins such as the polycomb repressive complex-2 protein EZH2, as well as other genes encoding DNA-binding proteins such as *Hmga1, Rorc, Ccdc62*, and *Zmym5*. Furthermore, we detected an A5SS event that showed sex-biased AS in *Miat/Gomafu*, a lncRNA that regulates splicing and represses transcription through association with the polycomb repressive complex (Barry et al., 2014; Zakutansky and Feng, 2022). One of the top 10 most significant AS events in young hippocampus was an A3SS splicing event in *Ift88*, a transport protein important for primary cilia maintenance in neurons (Tereshko et al., 2021). Indeed, in young but not aged hippocampus, we found sex-biased AS in a number of genes associated with primary cilia, including *Bbs4*, *Fuz*, *C2cd3*, *Rab34*, *Fam149b*, and *Cdk10*.

For genes on the X chromosome, we found sex bias in 10 splicing events in nine genes. For example, in the aged hippocampus, the X-chromosome gene *Huwe1*, encoding an E3 ubiquitin ligase linked to intellectual disability (Moortgat et al., 2018), was associated with an SE event that showed both a change with aging in females and a sex bias in the aged hippocampus. Overall, we found ~30% of genes with sex bias in AS also showed a change in splicing with aging (Figure 5C). Sex-specific aging-related alternative splicing events occurred in many of the genes mentioned above (e.g., *Ezh2*, *Rorc, Ift88*, *Fuz*, and *Cdk10*) and included isoform ratios that were specific to either young male or young female hippocampus (Figure 5D). Altogether, these results suggest that AS may contribute more to sex differences in the transcriptome than expression and that aging-related changes in AS are often sex-specific.

### Synaptic genes exhibit aging-dependent or sex-biased expression or AS in the hippocampus

Synaptic plasticity in the hippocampus is important for long-term memory formation (Bird and Burgess, 2008; Nabavi et al., 2014); therefore, we examined aging-dependent or sex-biased expression and splicing of synapse-related genes. Most of the synapse-related genes showed an aging-related decrease in expression in either male or female hippocampus and encode proteins critical for the excitatory postsynaptic terminal such as DLG4/PSD95, DLG2/PSD93, SYNGAP1, SHANK1, SHANK3, CACNG5, CAMK2A, and RGS14 (Table 1). Additionally, we found aging-related changes in the expression of genes regulating inhibitory neuron synaptic functions, including decreased expression of the inhibitory synaptogenic gene *Igsf21* (in females), the inhibitory synaptic adhesion gene *Igsf9b* (in both sexes), and calcium-binding protein gene *Calb1* (in both sexes) and increased expression of the calcium-binding protein gene *Pvalb* (in females) and inhibitory synaptic vesicle gene *Syt2* (in females) (Tanabe et al., 2017; Babaev et al., 2018). Together, these findings support previous reports of changing excitatory and inhibitory synaptic dynamics in the hippocampus with aging (McQuail et al., 2015; Rozycka and Liguz-Lecznar, 2017).

**Table 1:** Synapse-related DE genes.

Gene symbol	Gene name	DE	FDR
*Slc7a11/xCT*	Solute carrier family 7 (cationic amino acid transporter, y + system), member 11	Male-biased in aged	9.81E-07
*Ncan*	Neurocan	Downregulated with aging in males and females	2.75E-06 males, 1.54E-05 females
*Dlg4/Psd95*	Discs large MAGUK scaffold protein 4	Downregulated with aging in females	8.86E-06
*Cplx2*	Complexin 2	Downregulated with aging in males and females, male-biased in young adult and aged	1.29E-05 aging in females, 3.03E-03 aging in males, 3.36E-02 sex-biased in young, 1.28E-04 sex-biased in aged
*Apoe*	Apolipoprotein E	Upregulated with aging in males and females	1.41E-05 females, 4.32E-05 males
*Syngap1*	Synaptic Ras GTPase activating protein 1 homolog	Downregulated with aging in females	3.90E-05
*Spart/Spg20*	Spartin	Upregulated with aging in males and females	1.30E-04 females, 2.58E-03 males
*Ablim3*	Actin binding LIM protein family, member 3	Downregulated with aging in males and females	2.38E-04 females, 2.60E--03 males
*Camk2a*	Calcium/calmodulin-dependent protein kinase II alpha	Downregulated with aging in males and females	7.51E-04 females, 4.30E-02 males
*Igsf21*	Immunoglobulin superfamily, member 21	Downregulated with aging in females	1.79E-03
*Rgs14*	Regulator of G-protein signaling 14	Downregulated with aging in males	2.16E-03
*Marcks*	Myristoylated alanine rich protein kinase C substrate	Downregulated with aging in males	2.31E-03
*Adam11*	A disintegrin and metallopeptidase domain 11	Downregulated with aging in females	2.49E-03
*Grik5*	Glutamate receptor, ionotropic, kainate 5 (gamma 2)	Downregulated with aging in females	2.84E-03
*Musk*	Muscle, skeletal, receptor tyrosine kinase	Downregulated with aging in females	3.46E-03
*Cacna1g*	Calcium channel, voltage-dependent, T type, alpha 1G subunit	Downregulated with aging in females	3.85E-03
*Plppr4/ D3Bwg0562e*	Phospholipid phosphatase related 4	Downregulated with aging in males	4.07E-03
*Ddn*	Dendrin	Downregulated with aging in females	4.26E-03
*Cdh6*	Cadherin 6	Downregulated with aging in females	4.38E-03
*Ssh1*	Slingshot protein phosphatase 1	Downregulated with aging in females	4.73E-03
*Calb1*	Calbindin 1	Downregulated with aging in males and females	5.49E-03 females, 3.97E-02 males
*Pvalb*	Parvalbumin	Upregulated with aging in females	6.85E-03
*Shank3*	SH3 and multiple ankyrin repeat domains 3	Downregulated with aging in females, male-biased in aged	7.21E-03 aging, 3.77E-02 sex-biased
*Nlgn3*	Neuroligin 3	Downregulated with aging in males	7.56E-03
*Cntn2*	Contactin 2	Female-biased in aged	9.53E-03
*Ppt1*	Palmitoyl-protein thioesterase 1	Upregulated with aging in females	9.58E-03
*Igsf9b/ AI414108*	Immunoglobulin superfamily, member 9B	Downregulated with aging in males and females	9.89E-03 males, 4.13E-02 females
*Glra2*	Glycine receptor, alpha 2 subunit	Downregulated with aging in males and females	1.07E-02 females, 2.19E-02 males
*Begain*	Brain-enriched guanylate kinase-associated	Male-biased in aged	1.30E-02
*Syt2*	Synaptotagmin II	Upregulated with aging in females	1.35E-02
*Syndig1*	Synapse differentiation inducing 1	Downregulated with aging in males	1.54E-02
*Rab3c*	RAB3C, member RAS oncogene family	Male-biased in aged	1.74E-02
*Grm2*	Glutamate receptor, metabotropic 2	Downregulated with aging in females	1.90E-02
*Shank1*	SH3 and multiple ankyrin repeat domains 1	downregulated with aging in females	1.91E-02
*Dcdc2a*	Doublecortin domain containing 2a	Upregulated with aging in males	2.23E-02
*Cnih2*	Cornichon family AMPA receptor auxiliary protein 2	Downregulated with aging in females	2.76E-02
*Cacng5*	Calcium channel, voltage-dependent, L type, gamma subunit 5	Downregulated with aging in females	3.03E-02
*Prrt2*	Proline-rich transmembrane protein 2	Downregulated with aging in males	3.11E-02
*Dlg2/Psd93*	Discs large MAGUK scaffold protein 2	Downregulated with aging in males and females	3.31E-02 males, 4.69E-02 females
*Asic1*	Acid-sensing (proton-gated) ion channel 1	Downregulated with aging in females	3.34E-02
*Pclo*	Piccolo (presynaptic cytomatrix protein)	Upregulated with aging in females	3.67E-02
*Dpysl2*	Dihydropyrimidinase-like 2	Female-biased in aged	3.88E-02

Hippocampal aging-related or sex-biased genes associated with synaptic function.

Overall, we found significant AS events in a number of genes important for synaptic function (Table 2). One of the most significant sex-biased AS events in the aged hippocampus was the female-biased inclusion of exon 8a *Cacna1d*, a gene important for synaptic plasticity that encodes the pore-forming subunit of an L-type voltage-gated Ca^2+^ channel. Mutations in *Cacna1d* exon 8a have been implicated in autism spectrum disorder (Pinggera et al., 2015, 2017; Pinggera and Striessnig, 2016), and alternative splicing of exon 8 may contribute to channel voltage dependency differences (Hofer et al., 2021). We also found aging-related AS events in voltage-gated calcium channel genes *Cacna1c, Cacnb1*, and *Cacna1g* that may impact calcium channel function (Ernst and Noebels, 2009; Buraei and Yang, 2010; Lipscombe et al., 2013; Hu et al., 2017). We also detected sex-biased splicing (MXE in young, SE in aged) of *Mapk12*, a gene encoding the neuroprotective MAPK, p38γ, that is involved in regulating the post-synaptic density (Ittner et al., 2016; Asih et al., 2020). These results reveal that many synapse-related genes show sex-biased AS that may affect synaptic function.

**Table 2:** Synapse-related AS genes.

Gene		AS	AS event type	FDR
*Abi2*	Abl interactor 2	Aging in females	SE	2.19E-06
*Bcas1*	Brain enriched myelin-associated protein 1	Aging in females (two events), aging in males (two events)	SE	3.51E-05 and 2.25E-02 females, 2.91E-02 and 4.05E-02 males
*Cyfip1*	Cytoplasmic FMR1 interacting protein 1	Aging in males	SE	9.93E-05
*Cacna1d*	Calcium channel, voltage-dependent, L type, alpha 1D subunit	Sex bias in aged	SE	2.03E-04
*Src*	Rous sarcoma oncogene	Sex bias in aged (two events)	SE	6.07E-04 and 8.11E-03
*Mapk12*	Mitogen-Activated Protein Kinase 12	Sex bias in young adult and aged	MXE in young, SE in aged	8.46E-04 young, 1.26E-02 aged
*Gabra2*	Gamma-aminobutyric acid (GABA) A receptor, subunit alpha 2	Aging in males	SE	9.78E-04
*Srcin1*	SRC kinase signaling inhibitor 1	Sex bias in young adult, aging in males	SE	1.47E-03 sex bias, 5.66E-03 aging
*Myo6*	Myosin VI	Aging in females	SE	1.75E-03
*Adam23*	A disintegrin and metallopeptidase domain 23	Aging in males	SE	1.85E-03
*Ncam1*	Neural cell adhesion molecule 1	Aging in females	SE	2.39E-03
*Htr7*	5-hydroxytryptamine (serotonin) receptor 7	Aging in males	SE	2.85E-03
*Grin2c*	Glutamate receptor, ionotropic, NMDA2C (epsilon 3)	Aging in males	SE	5.76E-03
*Unc13b*	Unc-13 homolog B	Aging in males	SE	9.42E-03
*Cacna1c*	Calcium channel, voltage-dependent, L type, alpha 1C subunit	Aging in females	A5SS	1.26E-02
*Cacnb1*	Calcium channel, voltage-dependent, L type, beta 1 subunit	Aging in males	SE	1.83E-02
*Cacna1g*	calcium channel, voltage-dependent, T type, alpha 1G subunit	Aging in females	SE	2.20E-02
*Iqsec2*	IQ motif and Sec7 domain 2	Aging in males	SE	2.35E-02
*Dock10*	Dedicator of cytokinesis 10	Sex bias in young adult	A3SS	2.36E-02
*Unc13c*	Unc-13 homolog C	Sex bias in aged	SE	2.40E-02
*Nptn*	Neuroplastin	Sex bias in young adult	A3SS	2.45E-02
*Stxbp5*	Syntaxin binding protein 5 (tomosyn)	Aging in males	SE	2.91E-02
*Nrg1*	Neuregulin 1	Aging in females	MXE	3.46E-02
*Rims1*	Regulating synaptic membrane exocytosis 1	Aging in females	SE	3.50E-02
*Maoa*	Monoamine oxidase A	Sex bias in young adult	SE	3.50E-02
*Cadps*	Ca2 + −dependent secretion activator	Aging in males	A5SS	3.67E-02
*Cpne6*	Copine VI	Sex bias in aged	RI	3.98E-02
*Bin1*	Bridging integrator 1	Sex bias in young adult	SE	4.24E-02
*Grm7*	Glutamate receptor, metabotropic 7	Sex bias in aged	SE	4.41E-02

Alternative splicing events in the hippocampus in genes associated with synaptic function. SE = skipped exon, A5SS = alternative 5′ splice site, A3SS = alternative 3’splice site, MXE = mutually exclusive exons, RI = retained intron.

### Aging increases chromatin accessibility in the hippocampus of males and females

**Figure 6:**
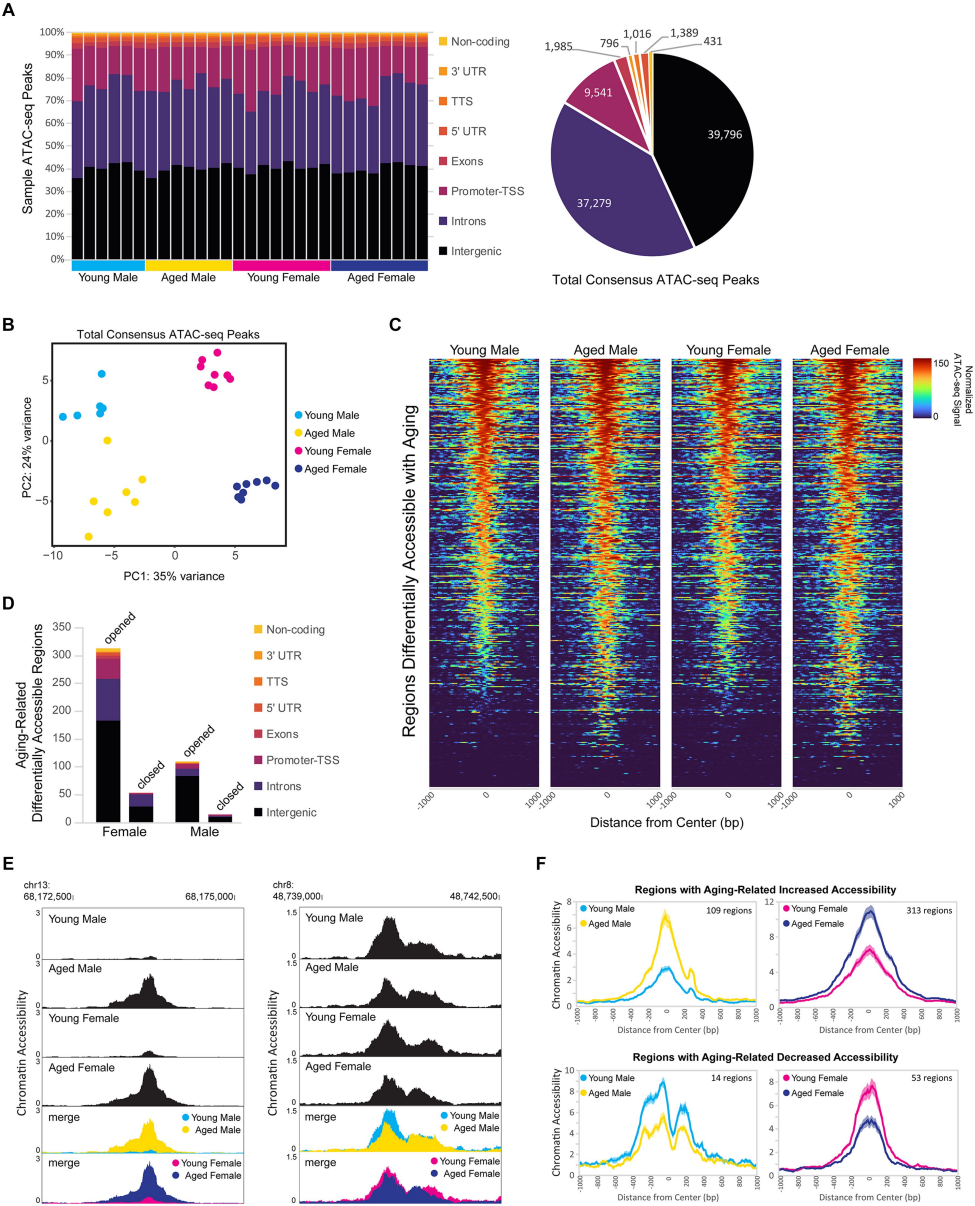
Aging is associated with higher chromatin accessibility, with most of the changes occurring in intergenic regions. **(A)** Genome annotation of individual sample ATAC-seq peaks, with each column representing a biological replicate (left) and genome annotation for total consensus ATAC-seq peaks (right). **(B)** Principle component analysis of ATAC-seq fragments in the total consensus peak set. **(C)** Heat maps of average normalized ATAC-seq signal per condition for aging-related differentially accessible regions (DARs). Normalized ATAC-seq signal/10 bp bin is shown for ± 1,000 bp around the ATAC-seq region center, and regions are sorted by young male ATAC signal in the ± 100 bp around the center. **(D)** Genome annotation of regions that were differentially accessible (FDR < 0.05) between young adult and aged hippocampus. **(E)** Browser track examples of regions showing aging-dependent differences in chromatin accessibility. Panel on the left shows the average normalized ATAC-seq signal from young and aged male and female hippocampus in the intergenic-MuRRS-int|LTR|ERV1 region chr13: 68173077-68174082 that was significantly more accessible in aged compared to young males and females (male aged vs. young log_2_FC = 3.85, FDR < 0.0001; aged vs. young log_2_FC = 3.00, FDR < 0.0001). Panel on the right shows the average normalized ATAC-seq signal in the intergenic region chr8: 48739982-48741266 that was significantly more accessible in young compared to aged males and females (male aged vs. young log_2_FC = −0.63, FDR = 0.04; female aged vs. young log_2_FC = −0.57, FDR =0.02). **(F)** ATAC-seq profiles for aging-related DARs in either males or females. Solid lines indicate the average of each condition’s normalized histogram (*n* = 6 young males, 7 aged males, 8 young females, and 8 aged females) with shading indicating s.e.m.

Dynamic changes in chromatin accessibility are critical to the precise control of gene expression patterns and have been shown to be altered with aging (Su et al., 2017; Zhang et al., 2022). We used ATAC-seq to probe for chromatin accessibility changes during aging in the hippocampus of male and female mice. The hippocampus of young adult and aged males and females showed a similar number of ATAC peaks, with a similar distribution of genome annotations: ~40% intergenic, 35% introns, and 18% promoter-TSS (Figure 6A; Supplementary Figure S3A). We analyzed a merged set of 92,233 replicated peaks (see Methods section; Figure 6A; Supplementary Figure S3B) and, similar to the results from our RNA-seq analysis, principle component analysis of fragment counts from ATAC-seq regions showed that samples were separated by sex and age by the first and second principle component, respectively (Figure 6B; Supplementary Figure S3C). Overall, 774 genomic regions were differentially accessible either with sex or aging (FDR < 0.05). There were 393 regions with sex-biased chromatin accessibility and 404 regions showing accessibility changes with aging (Figure 6C), including 23 regions that showed changes with both aging and sex. More differentially accessible regions (DARs) exhibited aging-related changes in female compared to male hippocampus (366 vs. 109 regions, chi-square test, *X^2^* = 139.4, *p* < 0.00001), and this result was consistent even when reduced numbers of female samples were included to match the number of male samples (Supplementary Figure S3D). Approximately 90% of aging-related DARs showed increased chromatin accessibility with aging, with intergenic regions making up the majority of DARs that either opened or closed with aging (Figure 6D). For example, an intergenic region in chromosome 13 was ~10 times more accessible in aged male and female hippocampus compared to the young adult hippocampus (Figure 6E left), whereas an intergenic region in chromosome 8 was ~1.5 times less accessible in aged compared to young adult hippocampus (Figure 6E right). On average, regions that opened with aging showed a 3.4- and 2.3-fold increase in accessibility with aging in males and females, respectively, and those regions that closed with aging showed a 1.8-fold aging-related decrease in accessibility in both males and females (Figure 6F). Altogether, these results indicate that aging leads to increased chromatin accessibility in the hippocampus, especially in non-coding regions.

**Figure 7:**
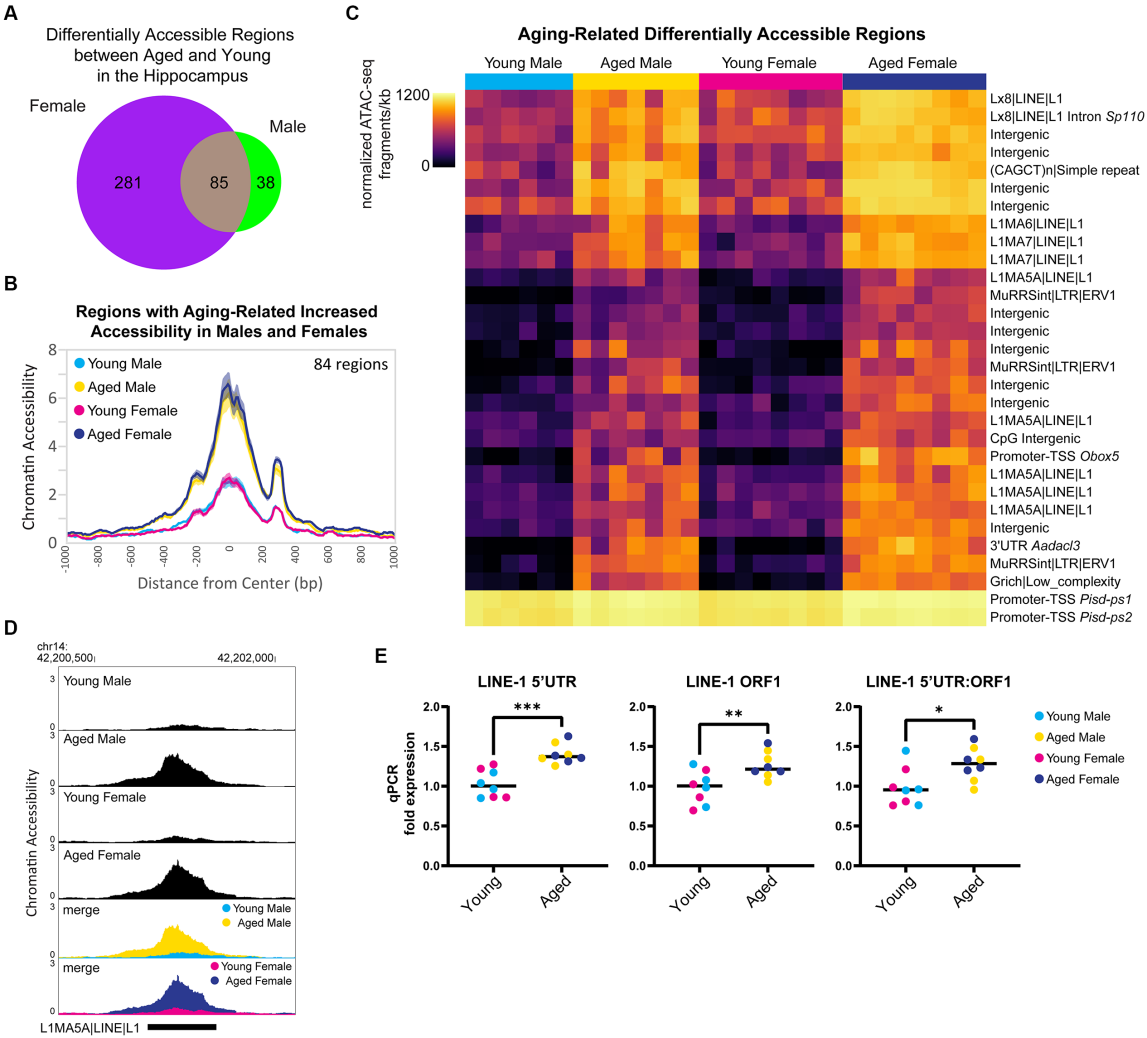
Aging is associated with higher accessibility in repetitive elements and higher expression of retrotransposon transcripts. **(A)** Venn diagram of the number of DARs (FDR < 0.05) in young adult and aged hippocampus. **(B)** ATAC-seq profiles for regions that showed increased accessibility with aging in both males and females. Solid lines indicate the average of each condition’s normalized histogram (*n* = 6 young males, 7 aged males, 8 young females, and 8 aged females) with shading indicating s.e.m. **(C)** Heat map of normalized total ATAC-seq signal per region for the top 30 aging-related DARs in both male and female hippocampus (top 30 by FDR, clustered by ATAC-seq signal). Each row represents a chromatin region with detailed annotation labeled on the right, and each column is a biological replicate. Normalized ATAC-seq fragments/kb are represented by color (color scale is non-linear, see Supplementary Table S3 for all normalized signal and log_2_FC values). **(D)** Browser track example of LINE-1 region showing aging-related increase in chromatin accessibility. Shown are the average normalized ATAC-seq signals from young and aged male and female hippocampus in the intergenic L1MA5A|LINE|L1 region chr14: 42200723-42201552 that was significantly more accessible with aging in males and females (aged vs. young male log_2_FC = 2.22, FDR < 0.0001; aged vs. young female log_2_FC = 2.05, FDR < 0.0001). **(E)** qPCR of LINE-1 transcript expression in young adult and aged hippocampus (*n* = four age/sex). LINE-1 5’UTR, LINE-1 ORF1, and LINE-1 5’UTR:ORF1 are three different primers for LINE-1 annealing to sequences within the 5’UTR, within ORF1, and spanning the 5’UTR and ORF1, respectively. Unpaired *t*-tests: LINE-1 5’UTR *p* < 0.001, LINE-1 ORF1 *p* = 0.009, LINE-1 5’UTR:ORF1 *p* = 0.02. * indicates *p* < 0.05, ** indicates *p* < 0.01, *** indicates *p* < 0.001.

First, we focused on the sex-independent chromatin accessibility changes in the hippocampus with aging. We found that 85 regions showed differential ATAC-seq signals with aging in both sexes (Figure 7A), and 99% of these regions exhibited aging-related increases in chromatin accessibility (84/85 regions opened, 1/85 closed). For the regions that gained accessibility in both sexes, there was ~3.8-fold increase in accessibility with aging (Figure 7B), and motif enrichment analysis showed that these regions were enriched for HOXA- and MEF2-like motifs (Supplementary Figure S4A). We ranked DARs by lowest FDR-adjusted *p*-value in either males or females and found that within the 30 most significant DARs, 13 were located in retrotransposable element-derived sequences, and all of these regions showed an opening in accessibility with aging (Figure 7C). For example, an intergenic L1MA5A-type LINE-1 region in chromosome 14 showed ~4-fold increase in accessibility in the hippocampus of aged animals of both sexes compared to young animals (Figure 7D). Twenty LINE-1 retrotransposon elements showed an aging-associated increase in accessibility in the hippocampus of both sexes (Supplementary Figure S4B). These regions encode truncated sequences incapable of retrotransposition; however, truncated LINE-1 sequences can be transcribed (Rangwala et al., 2009), leading to DNA damage (Kines et al., 2014) and immune activation (De Cecco et al., 2019; Simon et al., 2019). To test for an aging-related increase in the transcription of LINE-1 elements in the hippocampus, we performed qPCR for the retrotransposon LINE-1 with three different primer sets. We found that with aging, there was an average increase of 32% in LINE-1 transcript abundance in the hippocampus (LINE-1 5’UTR *p* < 0.001, LINE-1 ORF1 *p* < 0.01, LINE-1 5’UTR:ORF1 *p* < 0.05; Figure 7E), in agreement with the finding that LINE-ORF1 protein increased in the frontal cortex with aging (Zhang et al., 2022). Previous studies have found that LINE-1 retrotransposon activity is more common in neurons than other somatic cells and that it increases with aging (Reilly et al., 2013; Van Meter et al., 2014; Zhao et al., 2019; Gorbunova et al., 2021). We examined the chromatin accessibility of full-length intact LINE-1 genomic regions (Penzkofer et al., 2017) and found suppression of chromatin accessibility in these regions in both young adult and aged hippocampus (Supplementary Figure S4C), indicating that full-length LINE-1 regions as a whole remained repressed during aging. These results indicate that there is an opening of chromatin during aging, especially in retrotransposon-derived sequences, with an accompanying increase in LINE-1 transcript abundance, which may contribute to aging-related genome instability (Gorbunova et al., 2021).

### Sex differences in chromatin accessibility include male bias in promoter accessibility

**Figure 8:**
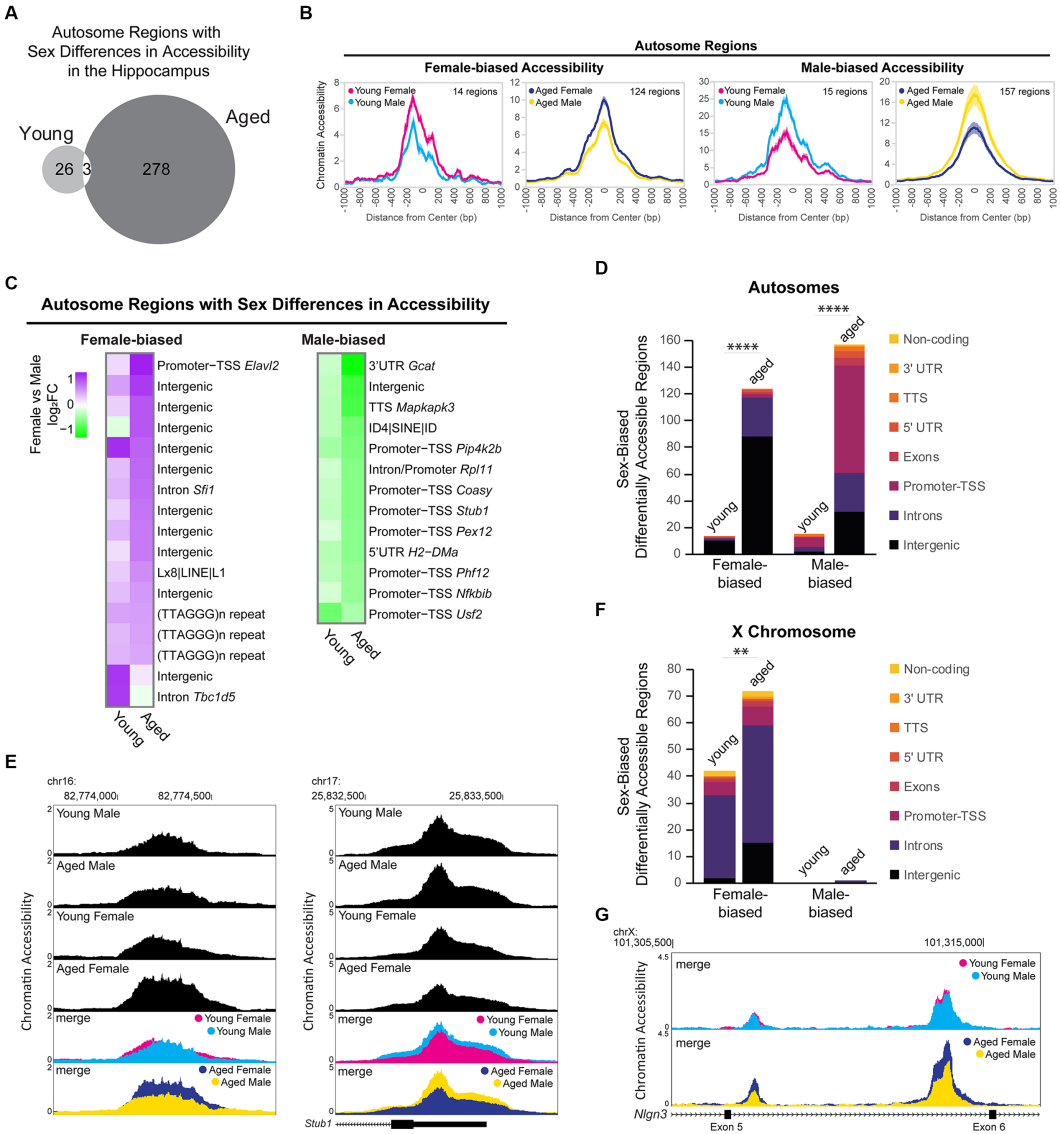
Sex differences in chromatin accessibility. **(A)** Venn diagram of the number of autosome sex-biased DARs in young adult and aged hippocampus (FDR < 0.05). **(B)** ATAC-seq profiles for sex-biased DARs on autosomes. Solid lines indicate the average of each condition’s normalized histogram (*n* = 6 young adult male, 7 aged male, 8 young adult female, 8 aged female) with shading indicating s.e.m. **(C)** Fold change heat map of sex-biased differentially accessibility for the top 30 autosome regions (top 30 by FDR, clustered by ATAC-seq signal; purple indicates higher accessibility in females and green indicates higher accessibility in males). Each row represents a chromatin region with detailed annotation labeled on the right. **(D)** Genome annotation of autosome regions that were differently accessible between female and male hippocampus. Chi-square test, young vs. aged *X^2^* = 87.7 more open female, *X^2^* = 117.3 more open male, *p* < 0.00001 (indicated with ****). **(E)** Browser track examples of regions showing sex differences in chromatin accessibility. Panel on the left shows the average normalized ATAC-seq signal from young adult and aged male and female hippocampus in the intergenic region chr16: 82773954-82774686 (1.5 Mb from nearest gene, *Ncam2*) that was significantly more accessible in aged females than males (FDR = 0.003, female vs. male log_2_FC = 0.79; one of two regions annotated to *Ncam2* showing female bias in accessibility). Panel on the right shows the average normalized ATAC-seq signal in the promoter-TSS region chr17: 25832563-25833564 of *Stub1* that was significantly more accessible in aged males than females (FDR = 0.002, female vs. male log_2_FC = −0.61). **(F)** Genome annotation of chromosome X regions that were differentially accessible between female and male hippocampus. Chi-square test, young vs. aged *X^2^* = 8.1 more open female, *p* < 0.01 (indicated with **). **(G)** Browser track example of X chromosome region showing sex differences in chromatin accessibility. The average normalized ATAC-seq signal is shown from young adult and aged male and female hippocampus in the intronic region chrX: 101308500-101316000 between exons 5 (ENSMUSE00001248461) and 6 (ENSMUSE00001264564) of *Nlgn3*. Two regions were significantly more accessible in aged females than males, with the second ATAC-seq peak also showing female-specific opening with aging (region chrX: 101309331-101309581 aged female vs. male log_2_FC = 0.88, FDR = 0.001; region chrX: 101313685-101314599 aged female vs. male log_2_FC = 0.85, FDR =1.19E-11; female aged vs. young log_2_FC = 0.70, FDR =1.11E-7).

To assess sex-dependent differences in chromatin accessibility in the hippocampus, we analyzed the DARs between males and females from young adult and aged mice. We identified 29 autosomal regions in young animals and 281 autosomal regions in aged animals that showed sex-biased differential accessibility, with aging associated with more sex differences in autosome chromatin accessibility (chi-square test, young vs. aged *X^2^* = 205.2, *p* < 0.00001; Figure 8A). Sex bias in chromatin accessibility resulted in ~1.7-fold change in peak ATAC-seq signal in sex-biased DARs (Figure 8B), relatively smaller than those seen with aging-related differential accessibility (Figure 6F). When we ranked sex-biased regions by lowest FDR in either young or aged animals, we found that the majority of the most significant male-biased regions were annotated to promoters, whereas the most significant female-biased regions were intergenic (Figure 8C). Similarly, when we analyzed the genomic ontology for all sex-biased DARs, we found that most regions that were more open in females versus males were annotated to intergenic regions (Figure 8D). In contrast, most male-biased accessible regions were promoter-TSS regions (8/15 in young, 80/157 in aged). For example, an intergenic region (1.5 Mb from nearest gene, *Ncam2*) was more accessible in aged females compared to aged males (Figure 8E left), and the TSS-promoter region of *Stub1* was more accessible in aged males compared to aged females (Figure 8E right). Motif analysis revealed that promoters with male-biased accessibility were enriched for CpG island-associated motifs, including CGCG/GFX and GC-box/SP/KLF motifs (Supplementary Figure S5A). Non-promoter regions with male-biased accessibility were enriched in CTCF, HOXA, ZNF382, and MEF2 motifs whereas non-promoter regions with female-biased accessibility were enriched for CTCF, E-box/NEUROD1, AP-1, and TLX motifs (Supplementary Figure S5B). Most mammalian promoter sequences are GC-rich and contain CpG islands (Yang et al., 2007). Indeed, we found the CpG content of accessible promoter regions in the hippocampus was ~7%, whereas that of accessible non-promoter regions was ~2% (Supplementary Figures S6A,B). However, we found that in the young and aged hippocampus, the CpG content in male-biased non-promoter regions was higher than average (~4%; Kruskal–Wallis tests with Dunn’s correction, *p* < 0.05; Supplementary Figure S6B). To test if the sex differences in accessibility for promoter and CpG-rich regions could be due to GC bias, we used smooth-quantile with GC-content normalization (Hicks et al., 2017; Van den Berge et al., 2022), which can correct for GC-content effects in ATAC-seq datasets. With this normalization technique, we found similar male bias in accessibility at promoters (QSmooth-GC; Supplementary Figure S6C) and CpG-rich regions (Supplementary Figures S6A,B; *p* < 0.0001); therefore, although we cannot rule out GC bias effects, these sex differences in accessibility appear to be biological in nature. Importantly, male-biased promoter accessibility did not appear to result in increased transcription at those genes, as none of the autosomal genes with male-biased accessibility at promoters showed sex-biased expression. Therefore, the male and female hippocampus may compensate for sex differences in promoter accessibility through different mechanisms to regulate expression.

We evaluated sex differences in chromatin accessibility of the X chromosome in young adult and aged hippocampus. Of the 2,595 ATAC-seq regions on the X chromosome, 74 regions (3%) showed sex differences in accessibility, with all but one region showing female-biased accessibility. We found 41 DARs on the X chromosome that showed a sex bias in both young and aged animals, all of which showed female-biased accessibility. Most of these regions were located within the *Dxz4* microsatellite and *Firre* locus important for X-chromosome 3D structure (29 female-biased regions in introns of *4933407K13Rik*, *Firre*, and *Gm35612/CrossFirre*). Many other female-biased regions were located in the gene body or promoter of female-biased genes (*Kdm6a, Ddx3x, Eif2s3x, Kdm5c*, and *Xist*) and/or genes located in the X inactivation center (*Xist*, *Ftx, Mir421, Jpx*, and *Gm9159*) (Yin et al., 2021). The number of regions with female-biased accessibility increased with aging (Figure 8F; chi-square test, young vs. aged *X^2^* = 8.1, *p* < 0.01), including aging-dependent female bias in accessibility in regions annotated to female-biased genes (*Kdm6a*, *Eif2s3x*, and *5530601H04Rik*) as well as in the gene bodies of the synaptic regulators *Nlgn3* and *Srpx2*. For example, two regions in an intron of *Nlgn3*, a gene that encodes the synaptic protein neuroligin-3 (Uchigashima et al., 2021) and whose expression was downregulated with aging in males, showed no sex bias in chromatin accessibility in young animals. However, a female-specific opening with aging resulted in female-biased accessibility in the aged hippocampus (Figure 8G). In the young hippocampus, all X chromosome regions showed a female bias in accessibility. Similarly, in the aged hippocampus, all X chromosome regions were more accessible in females than males, except for one region that showed a male bias in accessibility. This region spans the promoter, first exon, and intron of *Gm35612/CrossFirre*, a lncRNA that is repressed by the expression of *Firre*, which is a lncRNA involved in the maintenance of X chromosome inactivation (Fang et al., 2020). Altogether, these findings suggest that CpG-rich regions, including promoters, are more accessible in young and aged male compared to female hippocampus and that there is more divergence in chromatin accessibility in the hippocampus between males and females with aging.

### Aging and sex-biased DE is correlated with chromatin accessibility in associated regions and TSSs

**Figure 9:**
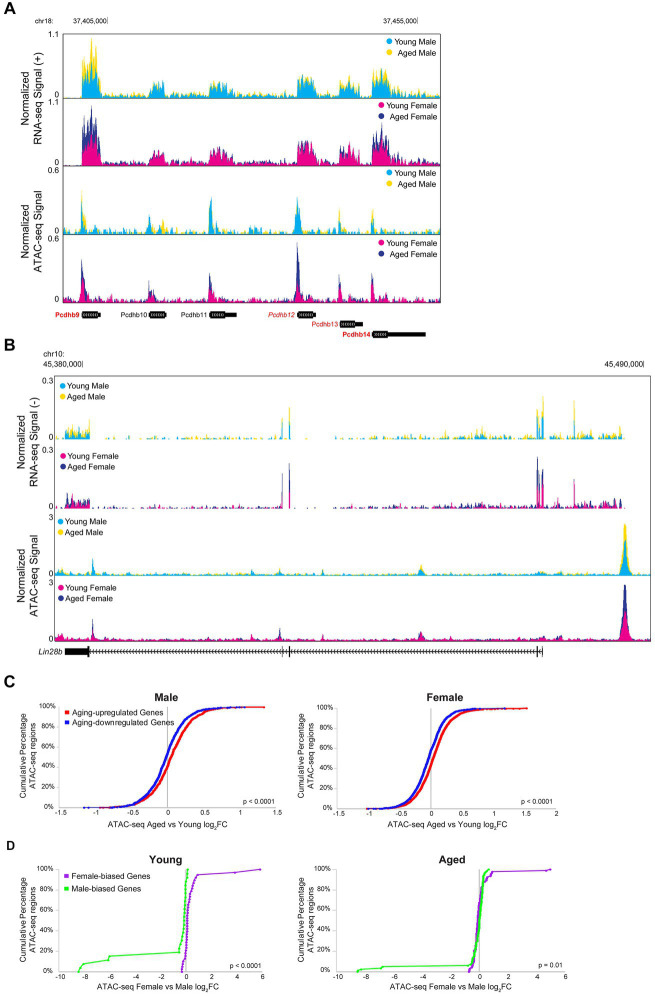
Aging and sex-biased differential expression is correlated with ATAC-seq differential accessibility. **(A)** Browser track example showing RNA-seq and ATAC-seq signal for a portion of the *Pcdhb* gene cluster (region chr18: 37397808-37458667), containing four genes that were significantly upregulated with aging. The top two tracks show the normalized plus-strand RNA-seq signal for the male (first row) and female (second row) hippocampus. The bottom two tracks show the normalized ATAC-seq signal for the male (third row) and female (fourth row) hippocampus. Genes that were upregulated with aging in both sexes are indicated with bold red (*Pcdhb9*: male aged vs. young adult log_2_FC = 0.82, FDR-adjusted *p* = 7.21E-33, female aged vs. young adult log_2_FC = 0.96, FDR-adjusted *p* = 1.68E-53; *Pcdhb14*: male aged vs. young adult log_2_FC = 0.46, FDR-adjusted *p* = 3.64E-6, female aged vs. young adult log_2_FC = 0.68, FDR-adjusted *p* = 6.29E-17). One gene was upregulated with aging in the female hippocampus only, indicated with italic red (*Pcdhb12*: male aged vs. young log_2_FC = 0.23, FDR-adjusted *p* = 0.24, female aged vs. young log_2_FC = 0.30, FDR-adjusted *p* = 0.02). One gene was upregulated with aging in the male hippocampus only, indicated with regular font red (*Pcdhb13*: male aged vs. young log_2_FC = 0.43, FDR-adjusted *p* = 0.006, female aged vs. young log_2_FC = 0.28, FDR-adjusted *p* = 0.07). Genes that were not DE with aging are indicated with black font. **(B)** Browser track example as in A, showing RNA-seq and ATAC-seq signal for the *Lin28b* locus (chr10: 45375000-45491000). RNA signal suggests a long *Lin28b* isoform, including additional exons present in the ENSMUST00000214555.1/Lin28b-202 transcript from an alternative promoter located at ATAC-seq peak. RNA upregulated with aging female: log_2_FC = 0.86, *p* = 4.9E-7; male: log_2_FC 0.82, *p* = 8.3E-5; ATAC opened with aging female: log_2_FC = 0.60, *p* = 2.2E-3; male: log_2_FC = 0.75, *p* = 2.2E-4. **(C)** Cumulative distributions of aging-related chromatin accessibility fold changes for male (left panel) and female (right panel) hippocampus for ATAC-seq regions annotated to DE genes that were either upregulated (red) or downregulated (blue) with aging (male: Kolmogorov–Smirnov D = 0.14, *p* < 0.0001; female: Kolmogorov–Smirnov D = 0.15, *p* < 0.0001). **(D)** Cumulative distributions of sex-biased chromatin accessibility fold changes for young (left panel) and aged (right panel) hippocampus for ATAC-seq regions annotated to DE genes that were either higher expressed in female (purple) or higher expressed in male (green) hippocampus (young: Kolmogorov–Smirnov D = 0.68, *p* < 0.0001; aged: Kolmogorov–Smirnov D = 0.24, *p* = 0.01).

Transcription is a dynamic process involving the controlled coordination of regulatory elements such as promoters and enhancers, and DNA accessibility can indicate active regulatory elements bound by or permissive to chromatin binding factors (Cramer, 2019; Andersson and Sandelin, 2020). Therefore, we wanted to assess the relationship between aging- or sex-related changes in chromatin accessibility and gene expression. For example, many of the genes in the *Pcdhb* gene cluster were upregulated with aging, and a corresponding increase in chromatin accessibility can be observed in the ATAC-seq signal in this region (Figure 9A). Similarly, *Lin28b*, a gene encoding an RNA-binding protein that regulates miRNA maturation was upregulated with aging in the hippocampus of males and females, and a region upstream of *Lin28b* showed an aging-related increase in accessibility in the hippocampus of both sexes (RNA FDR < 0.001 and ATAC peak on far right, upstream/alternative promoter of *Lin28b*, FDR < 0.01 for both sexes; Figure 9B).

To compare RNA-seq and ATAC-seq results across all DE genes, we first assessed the relationship between aging or sex bias fold changes in expression with the fold changes in chromatin accessibility for ATAC-seq regions closest to the TSS of those genes. We found that aging-related changes in gene expression were significantly correlated with aging-related changes in chromatin accessibility in the male and female hippocampus (male: of 370 DE genes, 316 genes were associated with 2,044 ATAC-seq regions, Spearman r_s_ = 0.12, *p* < 0.0001; female: of 707 DE genes, 596 were associated with 3,600 regions, Spearman r_s_ = 0.15, *p* < 0.0001), and a stronger correlation was observed when including only ATAC-seq regions that were within 10 kb of the TSS (male Spearman r_s_ = 0.26, *p* < 0.0001, female Spearman r_s_ = 0.27, *p* < 0.0001). Similarly, ATAC-seq regions closest to the TSS of genes that were upregulated with aging in either male or female hippocampus showed more aging-related opening compared to ATAC-seq regions closest to the TSS of genes that were downregulated with aging (Figure 9C; male: Kolmogorov–Smirnov D = 0.14, *p* < 0.0001; female: Kolmogorov–Smirnov D = 0.15, *p* < 0.0001).

We also found a correlation between sex bias in expression and chromatin accessibility in the young hippocampus (young: of 17 DE genes, 16 were associated with 66 ATAC-seq regions, Spearman r_s_ = 0.74, *p* < 0.0001; aged: of 42 genes, 34 were associated with 178 ATAC-seq regions, Spearman r_s_ = 0.00, *p* = 0.96), and we found significant correlations in both young and aged hippocampus for regions within 10 kb of the TSS (young Spearman r_s_ = 0.88, *p* < 0.0001, aged Spearman r_s_ = 0.42, *p* < 0.01). We specifically examined the ATAC-seq regions annotated to the female-biased myelin-related genes from Figure 3D, and found that although some regions showed a trend of female-biased accessibility in the aged hippocampus, many did not, and none showed a significant sex bias (Supplementary Figure S7A). However, overall, ATAC-seq regions closest to the TSS of female- versus male-biased genes showed higher chromatin accessibility in female versus male hippocampus in both young and aged animals (Figure 9D; young: Kolmogorov–Smirnov D = 0.68, *p* < 0.0001; aged: Kolmogorov–Smirnov D = 0.24, *p* = 0.01).

To evaluate the relationship of aging and sex bias DE with chromatin accessibility independent of consensus peaks, we analyzed the ATAC-seq signal surrounding DE genes’ TSSs. We found that overall chromatin accessibility increased in the region surrounding the TSS of genes that were upregulated with aging in the female hippocampus (Supplementary Figure S7B; female: Kolmogorov–Smirnov *p* = 0.03, male: *p* = 0.11). Furthermore, chromatin accessibility was higher in the female hippocampus in regions surrounding the TSS of female-biased genes, and chromatin accessibility was higher in males in regions surrounding the TSS of male-biased genes (Supplementary Figure S7C; female-biased expression, young: Kolmogorov–Smirnov *p* = 0.003; aged: *p* < 0.0001; male-biased, young: *p* < 0.0001; aged: *p* = 0.0001). In summary, we found that changes in expression tended to correlate with changes in chromatin accessibility, although we note that these correlations may be underestimated, as enhancers often do not regulate the gene with the nearest TSS (Andersson et al., 2014; Furlong and Levine, 2018).

## Discussion

Memory impairments are a hallmark of aging. In both humans and rodents, the hippocampus is important for long-term memory formation (Bird and Burgess, 2008; Morrison and Baxter, 2012; Allen and Fortin, 2013), and like humans, mice show memory impairments and changes in hippocampal function with aging (Benice et al., 2006; von Bohlen und Halbach et al., 2006; Peleg et al., 2010; Wimmer et al., 2012; Stilling et al., 2014; Pereda et al., 2019). While cell type abundance in the brain, including the hippocampus, does not appear to change with aging (Long et al., 1998; Stilling et al., 2014; Ximerakis et al., 2019; Hahn et al., 2023), a number of cellular processes, including chronic inflammation, dysregulated proteostasis, synaptic function, retrotransposon activation, and genomic instability, are altered with aging and can impact cognitive function and memory formation (Morrison and Baxter, 2012; Pal and Tyler, 2016; Gorbunova et al., 2021; Simpson and Chandra, 2021; López-Otín et al., 2023). Although there are sex differences in aging-related memory deficits and Alzheimer’s disease (Gao et al., 1998; Herlitz and Rehnman, 2008; Jack et al., 2015; McCarrey et al., 2016; Beam et al., 2018; Anstey et al., 2021; Davis et al., 2021), this is the first study, to the best of our knowledge, that examines how aging-related changes in alternative splicing and chromatin accessibility in the hippocampus differ between males and females.

Using a genome-wide approach to understand how aging and sex affect the transcriptome and chromatin accessibility in the mouse hippocampus, our results provide a framework of the molecular mechanisms that may contribute to aging-related memory impairment. Our findings support a number of themes that are consistent with existing hypotheses of aging, including aging-related inflammation, changes in myelination, synaptic alterations, calcium dysregulation, loss of heterochromatin, and increased retrotransposon activity (Morrison and Baxter, 2012; Nikoletopoulou and Tavernarakis, 2012; Mosher and Wyss-Coray, 2014; Pal and Tyler, 2016; Gorbunova et al., 2021; Simpson and Chandra, 2021; Franklin and Simons, 2022; López-Otín et al., 2023). Our results also, however, reveal significant sex differences in the transcriptome and chromatin environment in the hippocampus and further show that aging amplifies sex bias in expression and chromatin accessibility.

### Aging-related changes in immune genes

Immune response genes were upregulated with aging in both male and female hippocampus, in line with numerous reports of chronic inflammation, upregulation of immune genes, and activation of microglia with aging in the hippocampus and other brain regions (Sheng et al., 1998; Berchtold et al., 2008; Cribbs et al., 2012; Mosher and Wyss-Coray, 2014; Stilling et al., 2014; Mangold et al., 2017; Spittau, 2017; Allen et al., 2023; Hahn et al., 2023). We found more changes in gene expression overall with aging in the female hippocampus than in the male hippocampus (Figure 1D), a result that is consistent with previous microarray studies in both human and mouse hippocampus (Berchtold et al., 2008; Cribbs et al., 2012; Mangold et al., 2017), but that differs from other brain regions (Berchtold et al., 2008). In agreement with the fact that females mount stronger immune responses than males (Klein and Flanagan, 2016; Gal-Oz et al., 2019), we found that female hippocampus showed larger aging-related changes in expression of immune-response genes (e.g., *C4b*, Figure 2D). Given that the hippocampus has been reported to be more immune-alert than other brain regions (Grabert et al., 2016), the greater aging-related changes in gene expression in female hippocampus may result in part from higher female immune response.

### Aging-related changes in myelin sheath genes

We detected significant aging-dependent changes in the expression and alternative splicing of myelin sheath genes. Produced by oligodendrocytes, myelin is important for axon integrity and action potential conductance (Nave and Werner, 2014) and has been found to be critical for memory retention (Xin and Chan, 2020). During aging, impaired myelin debris clearance and diminished OPC differentiation reduce the efficiency of remyelination (Sim et al., 2002; Franklin and Goldman, 2015; Franklin and Simons, 2022; Chapman et al., 2023). A previous study showed that enhancing OPC proliferation and myelination in the aged hippocampus increased memory retention in mice (Iram et al., 2022). We detected aging-dependent female bias in the expression of genes encoding myelin sheath components, including *Mag*, *Mbp*, *Mog*, and *Mal* (Figure 3D). The compact myelin gene, *Mbp*, was upregulated with aging in female but not male hippocampus by RNA-seq and qPCR, although MBP protein did not show sex bias in overall abundance (Figure 4C). There were, however, sex differences in the abundance of specific MBP isoforms in the aged hippocampus (Figure 4C; Supplementary Figures S2D,E), indicating that male and female hippocampus show differential regulation of aging-related *Mbp* transcript and protein isoform expression. Altogether, the female-biased expression of myelin sheath-related genes with aging could suggest that female hippocampus undergoes less myelin degeneration or more remyelination than male hippocampus during aging, consistent with studies in aged rats showing a female bias in remyelination and white matter volume (Li et al., 2006; Yang et al., 2008). Indeed, the female-biased aging-related upregulation of *Bcas1*, a marker of early actively myelinating oligodendrocytes (Fard et al., 2017), could reflect more active remyelination in aged female versus male hippocampus. Because an immune response is required for myelin debris clearance and OPC recruitment (Peters, 2002; Franklin and Simons, 2022), one possibility is that a higher immune response in the female hippocampus may lead to more efficient myelin debris clearance and OPC recruitment necessary for remyelination.

We found aging-related splicing differences not only in *Mbp* but also in myelin sheath genes *Mag* and *Bcas1* in male and female hippocampus (Figure 4B). The alternative splicing of *Mag*, which encodes a myelin-membrane protein, into large (L-MAG), exon 12-skipped, and small (S-MAG), exon 12-included, isoforms is tightly regulated during development, with L-MAG predominating during early myelinogenesis with potentially larger myelination capacity and S-MAG expression increasing during maturation to maintain myelination (Tropak et al., 1988; Quarles, 2007). Our finding that aging resulted in an increase in S-MAG and a decrease in L-MAG isoforms could point to aging-related reduced myelin formation or capacity. Similarly, the alternative splicing of exon 2 in *Mbp* is also known to be regulated during development (Harauz and Boggs, 2013; Müller et al., 2013). MBP isoforms containing exon 2 are enriched during active myelin formation, whereas isoforms excluding exon 2 are predominately expressed in adulthood (Woodruff and Franklin, 1998; Li et al., 2000). These isoforms differ in their subcellular location and function, with exon 2-containing isoforms localized to the cytosol and nucleus, where they regulate myelin development and differentiation, whereas exon 2-excluding isoforms are localized to the oligodendrocyte myelin membrane, where they are essential for the formation and function of compact myelin (Harauz and Boggs, 2013). In rats, loss of exon 2-included isoforms (but not other MBP isoforms) with aging was associated with split and retracted myelin sheaths, especially in paranodal regions (Sugiyama et al., 2002). Our finding that exon 2-containing MBP isoform expression decreased with aging suggests that this decrease may result in the loss of molecular signaling pathways regulating remyelination and maintenance of the axo-glial junction. Less is known about alternative splicing of the myelination gene, *Bcas1;* however, aging-related splicing of *Bcas1* has been previously reported in the mouse hippocampus (sex not specified) (Stilling et al., 2014). Notably, in oligodendrocytes, the alternative splicing of *Mag*, *Mbp*, and *Bcas1* is regulated by the RNA-binding protein quaking (Wu et al., 2002; Zhao et al., 2010; Darbelli et al., 2017). Although we did not detect aging-related differential expression or alternative splicing of the quaking gene (*Qk/Qki*), phosphorylation of quaking can regulate its binding to and stabilization of *Mbp* RNA (Zhang et al., 2003). Altogether, it is possible that altered quaking function during aging in the hippocampus may lead to aging-related changes in alternative splicing of *Mag*, *Mbp*, and *Bcas1*, contributing to the decrease in myelination capacity observed with aging.

### Aging-related changes in synaptic function genes

Previous studies have shown that in the hippocampus, aging is associated with alternations in synaptic function, including deficits in synaptic plasticity induction, reduction in postsynaptic density size, and dysregulated calcium currents (Shankar et al., 1998; Kumar et al., 2009; Morrison and Baxter, 2012). In alignment with these studies, we detected changes in the expression and alternative splicing of genes regulating synaptic function with aging in the hippocampus of male and female mice (Tables 1, 2). We found aging-related changes in the expression of genes encoding both inhibitory and excitatory synapse-related proteins, as well as changes in calcium buffer and calcium channel genes. We found several L- and T-type calcium channel subunit genes that were either downregulated with aging or showed aging-related changes in alternative splicing. Calcium is required for long-lasting synaptic plasticity in the hippocampus and is tightly controlled in neurons through the regulation of calcium buffers and calcium release from internal stores, as well as calcium influx through NMDA receptors and voltage-dependent calcium channels (Nikoletopoulou and Tavernarakis, 2012). Dysregulated calcium homeostasis not only suppresses synaptic plasticity but also causes neurons to be more vulnerable to damage from oxidative stress, and previous studies have shown that aging results in increased L-type voltage-dependent calcium currents that disrupt long-lasting synaptic plasticity and memory in the hippocampus of rodents (Kumar et al., 2009; Nikoletopoulou and Tavernarakis, 2012; Oh et al., 2013; Pereda et al., 2019). Additional genes important for synaptic plasticity and memory, including *Camk2a* (Elgersma et al., 2002), *Ablim3* (Guo et al., 2018), and *Cplx2*, were downregulated with aging in both sexes. Furthermore, male and female hippocampus showed different aging-related changes in expression and alternative splicing, including male-biased expression of the cysteine/glutamate antiporter gene *Slc7a11/xCT*, whose deletion in male mice has been shown to improve aging-related memory deficits (Verbruggen et al., 2022).

### Sex differences in gene expression and splicing

Sex-biased expression and splicing were observed in both young and aged hippocampus. Sex differences in the brain can result from the influence of gonadal sex hormones and sex chromosome-expressed genes. In both humans and rodents, circulating and brain-derived estrogens as well as circulating androgens diminish with aging, and their loss is linked to memory impairment (Khosla et al., 1998; Edinger and Frye, 2007; Cai and Li, 2020; Low et al., 2020; Taxier et al., 2020). We found that relatively few genes showed sex-biased expression in the young adult hippocampus (Figure 3A), and most were sex chromosome-expressed genes. Two notable exceptions were the male-biased expression of the synaptic vesicle gene, complexin 2 (*Cplx2*), and the female-biased expression of the neuropeptide gene, *Npy. Npy* has been shown to be upregulated by estradiol in the hippocampus (Ledoux et al., 2009), and correspondingly, we found female-biased expression in the young adult hippocampus but aging-related downregulation in the female hippocampus. Sex chromosome-expressed genes themselves can also contribute to downstream sex-biased expression. For example, the X chromosome-expressed genes *Kdm6a/Utx* and *Kdm5c* are histone demethylases that show female-biased expression, regulate transcription in the brain, and show incomplete functional overlap with their Y-chromosome paralogs *Uty* and *Kdm5d* (Iwase et al., 2016; Scandaglia et al., 2017; Tang et al., 2017; Davis et al., 2020; Cabrera Zapata et al., 2022). Therefore, sex hormones and sex chromosome-expressed genes both likely influence the sex-biased expression of hippocampal genes we observe.

Sex-biased alternative splicing can also lead to downstream sex bias in the transcriptome of the hippocampus. We found sex-biased alternative splicing of *Hmga1* with male-biased inclusion of the splice isoform corresponding to long exon-containing *Hmga1a* over the short exon-containing *Hmga1b* (Figure 5B). HMGA1 is a chromatin modifier and splicing protein whose binding results in more open chromatin (Benecke and Eilebrecht, 2015; Sumter et al., 2016). While the function of the different splice isoforms in chromatin accessibility is not understood, a previous study in female human cell lines has shown that *Hmga1a*, but not *Hmga1b*, regulates the splicing pattern of presenilin-2 seen in sporadic Alzheimer’s disease (Manabe et al., 2003). HMGA1a has also been shown to regulate the splicing and DNA binding of estrogen receptor α in breast cancer cells (Massaad-Massade et al., 2002; Ohe et al., 2018). Another gene known to regulate splicing, *Miat/Gomafu*, also exhibited sex-biased alternative splicing in the young adult hippocampus (Figure 5B). *Gomafu* expression has been shown to be regulated by estrogen in breast cancer cells (Li et al., 2018) and by synaptic activity in neurons (Barry et al., 2014; Zakutansky and Feng, 2022), and although many alternatively spliced *Gomafu* isoforms have been identified, little is known about their distinct functions (Zakutansky and Feng, 2022). Therefore, the interplay of sex hormones and sex-biased expression and alternative splicing of regulatory factors may contribute to downstream sex-biased patterns in the hippocampus.

### Aging-related changes in chromatin accessibility

This is the first study, to the best of our knowledge, that examines sex differences in chromatin accessibility with aging in the hippocampus. Our finding that chromatin structure is more open with aging in both sexes (Figures 6C,D) is consistent with previous findings in male hippocampal neurons (Zhang et al., 2022) and with the “heterochromatin loss” model of aging (Villeponteau, 1997; Tsurumi and Li, 2012; Pal and Tyler, 2016). This model suggests that there is a loss of heterochromatin during aging, leading to alterations in the expression of genes residing in these regions (Villeponteau, 1997). Chromatin accessibility is associated with active regulatory elements and is regulated by chromatin and histone modifiers. Histone variants and histone post-translational modifications are associated with open or closed chromatin, and, in general, CpG island methylation is correlated with decreased chromatin accessibility (Collings and Anderson, 2017; Cramer, 2019; Andersson and Sandelin, 2020). Changes in the expression of histone proteins, as well as altered histone modifications and DNA hypo- or hyper-methylation, are widely documented to occur during aging; indeed, aging-related changes in DNA methylation serve as the basis of epigenetic clocks (Horvath et al., 2016; Pal and Tyler, 2016; Simpson and Chandra, 2021; Lu et al., 2023). Therefore, loss of histone proteins, loss of repressive histone marks, and changes in DNA methylation likely contribute to the aging-related opening in chromatin accessibility we detected in the male and female hippocampus.

Our results show that most of the aging-related increases in chromatin accessibility occurred in intergenic and intronic regions, many of which contained retrotransposon LINE-1-derived sequences (Figure 7C). Furthermore, we found increased expression of LINE-1 transcripts with aging in the hippocampus (Figure 7E). Increased retrotransposon activity has been reported to accompany aging in both humans and rodents, and the brain has been reported to exhibit more retrotransposon activity than other tissues (Barter and Foster, 2018; Zhou and Smith, 2019; Gorbunova et al., 2021; Zhang et al., 2022). In young mammalian cells, the histone deacetylase SIRT6 binds to LINE-1 retrotransposon sequences and represses their activity by modulating the heterochromatin status of nearby regions. However, during aging, SIRT6 is depleted from LINE-1 regions, and histone repressive marks and CpG methylation are lost in transposable element regions, leading to the expression of LINE-1 transcripts and the accompanying DNA damage and cellular immune activation. Furthermore, repression of aging-related retrotransposon activity through overexpression of SIRT6 has been shown to increase lifespan (Van Meter et al., 2014; Pal and Tyler, 2016; Gorbunova et al., 2021). We found that full-length intact LINE-1 regions in both male and female hippocampus remained robustly inaccessible with aging (Supplementary Figure S4C), suggesting that transposition-capable sequences as a whole remain suppressed, although individual instances of full-length LINE-1 element activity may occur. LINE-1 derived sequences that opened with aging were truncated sequences incapable of retrotransposition; however, the expression of truncated LINE-1 transcripts has been reported to cause cellular inflammation and DNA damage (Kines et al., 2014; De Cecco et al., 2019; Simon et al., 2019). Thus, loss of repressive histone marks and CpG methylation at LINE-1 regions likely contributed to the aging-related opening of chromatin at those regions and to the increased LINE-1 expression we observed in the male and female hippocampus.

### Sex differences in chromatin accessibility

We found that male and female hippocampus showed sex differences in chromatin accessibility on autosomes, and the number of regions with sex-biased accessibility increased with aging (Figure 8D). These differences could be a result of sex-biased expression or activity of histone and/or DNA methylation modifiers. Sex hormones have been shown to affect DNA methylation in human blood (Harbs et al., 2023) and chromatin accessibility in the female ventral hippocampus of young adult mice (Jaric et al., 2019). We detected few sex differences in chromatin accessibility in the young adult hippocampus (Figure 8A); however, differences in aging-related DNA methylation between sexes could contribute to the sex-biased accessibility observed in the aged hippocampus. Indeed, it has been reported that aging-related DNA methylation changes differed between male and female hippocampus in mice (Masser et al., 2017). Furthermore, studies in human blood cells have shown aging-related sex differences in DNA methylation (Li X. et al., 2020; Yusipov et al., 2020; Vershinina et al., 2021). We found male-biased chromatin accessibility in a subset of promoters and CpG-rich regions (Figures 8C,D; Supplementary Figure S6), raising the possibility that CpGs in these regions could be hypermethylated in female compared to male hippocampus. We did not detect sex bias in the expression of DNA methylation-related proteins; therefore, DNA methylation patterns established in development, or other mechanisms regulating the activity of DNA methylation-related proteins, may contribute to these differences in accessibility.

Alternatively, or in conjunction with sex-biased DNA methylation, sex differences in histone modifications may contribute to the male-biased accessibility in promoter regions we observed. As mentioned previously, we detected female-biased expression of the X chromosome-linked histone modifiers *Kdm6a* and *Kdm5c*. *Kdm5c* is a histone demethylase that removes methyl groups from H3K4me2/3 (Brookes et al., 2015; Scandaglia et al., 2017; Ugur et al., 2023). H3K4me3 is a histone mark associated with active promoters, and *Kdm5c* has been found to reduce H3K4 trimethylation at promoters and suppress transcription (Iwase et al., 2016; Scandaglia et al., 2017; Link et al., 2020). *Kdm5c* escapes X-inactivation and has been shown to exhibit female-biased expression in the brain (Xu et al., 2008). We found that *Kdm5c* showed female-biased expression in both young adult and aged hippocampus. In young adult and aged males, the Y-chromosome paralog, *Kdm5d*, was expressed at slightly lower levels than *Kdm5c*. Although we found the summed *Kdm5c/d* expression in males is higher than *Kdm5c* expression in the female hippocampus, *Kdm5d* is unable to compensate for *Kdm5c* function in the brain (Samanta et al., 2022). Indeed, loss-of-function mutations in *Kdm5c* reduce H3K4me2/3 demethylation in a subset of promoters and enhancers and have been shown to lead to a form of X-linked intellectual disability in males (Jensen et al., 2005; Brookes et al., 2015). *Kdm5c* shows preferential binding to promoters, especially those containing CpG islands (Iwase et al., 2016), and we found male-biased accessibility at promoters and CpG-rich regions. Therefore, one possibility is that female-biased expression of *Kdm5c* may result in greater demethylation of H3K4me3 at promoters in the female hippocampus and contribute to lower chromatin accessibility in those regions. A previous study found promoter CpG sites with reduced DNA methylation in the blood of male patients with X-linked intellectual disability and mutations in *Kdm5c* (Grafodatskaya et al., 2013). Furthermore, the authors showed that these promoter regions also showed female-biased DNA hypermethylation in the brain of controls, as well as DNA hypermethylation that correlated with *Kdm5c*/*Kdm5d* dosage (Grafodatskaya et al., 2013). Therefore, female-biased *Kdm5c* expression may lead to greater histone demethylation and DNA methylation at promoters in females, thereby contributing to the male bias in promoter accessibility we observed. Notably, the male bias in promoter accessibility is not sufficient to drive sex-biased expression of the associated genes. Therefore, other mechanisms may compensate for sex differences in promoter accessibility to regulate expression.

In conclusion, this study provides a comprehensive map of gene expression, splicing, and chromatin accessibility changes in the hippocampus during aging in both male and female mice. Our findings of sex-biased alternative splicing and aging-dependent sex bias in expression and chromatin accessibility illustrate the importance of including female animals in studies of brain aging. These findings also provide novel starting points for future research on chromatin structure and transcriptomics in the hippocampus that can guide treatments for aging-related memory decline.

## Data availability statement

The datasets presented in this study can be found in online repositories. The names of the repository/repositories and accession number(s) can be found in the article/Supplementary material.

## Ethics statement

The animal study was approved by UCLA Institutional Animal Care and Use Committee. The study was conducted in accordance with the local legislation and institutional requirements.

## Author contributions

JA: Conceptualization, Data curation, Formal analysis, Investigation, Methodology, Project administration, Visualization, Writing – original draft, Writing – review & editing. YT: Conceptualization, Funding acquisition, Investigation, Methodology, Validation, Writing – original draft. FG: Formal analysis, Visualization, Writing – review & editing. C-HL: Formal analysis, Writing – review & editing. MW: Investigation, Validation, Visualization, Writing – review & editing. SN: Methodology, Writing – review & editing. GC: Funding acquisition, Supervision, Writing – review & editing. DB: Funding acquisition, Supervision, Writing – review & editing. KM: Conceptualization, Funding acquisition, Project administration, Supervision, Writing – review & editing.

## Footnotes

^1^ https://www.ncbi.nlm.nih.gov/geo/query/acc.cgi?acc=GSE244506

^2^ https://github.com/vsbuffalo/scythe/

^3^ http://genome.ucsc.edu

^4^ https://www.ncbi.nlm.nih.gov/geo/query/acc.cgi?acc=GSE244506

^5^ http://pga.mgh.harvard.edu/primerbank/
