# Supplementary material for: Aging differentially alters the transcriptome and landscape of chromatin accessibility in the male and female mouse hippocampus
Source: Front Mol Neurosci. 2024 Jan 22;17:1334862. doi: 10.3389/fnmol.2024.1334862 (PMC10839115; doi:10.3389/fnmol.2024.1334862)
Supplement: Supplementary file 4 [file Data_Sheet_1.pdf]

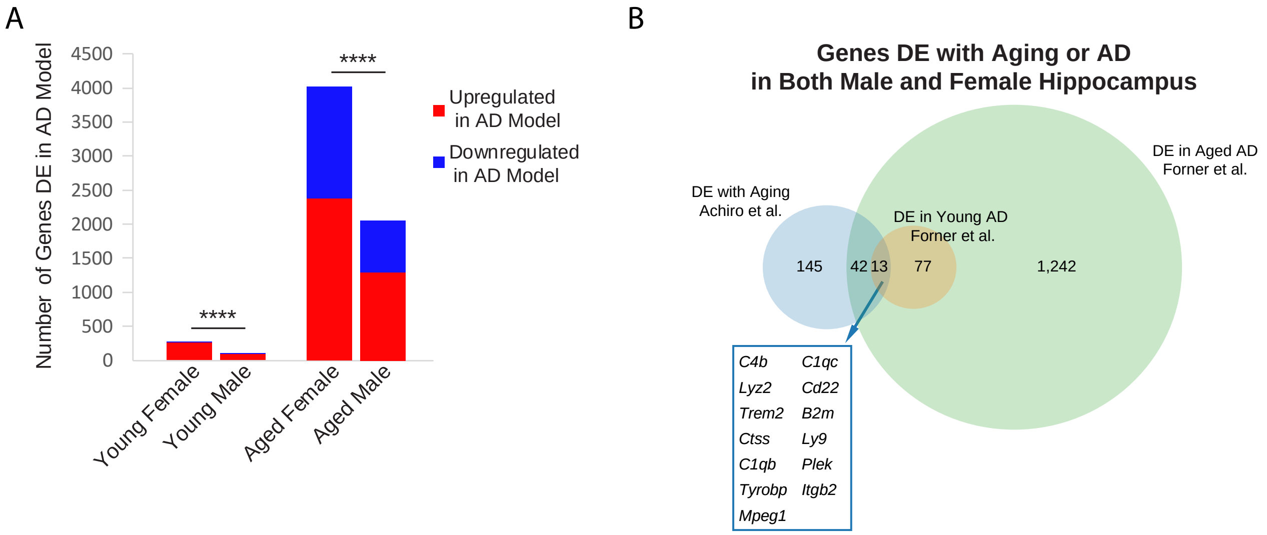
A
Number of Genes DE in AD Model
4500
4000
3500
3000
2500
2000
1500
1000
500
0
****
****
Young Female
Young Male
Aged Female
Aged Male
Upregulated in AD Model
Downregulated in AD Model
B
Genes DE with Aging or AD in Both Male and Female Hippocampus
DE with Aging Achiro et al.
DE in Young AD Forner et al.
DE in Aged AD Forner et al.
145
42
13
77
1,242
C4b
C1qc
Lyz2
Cd22
Trem2
B2m
Ctss
Ly9
C1qb
Plek
Tyrobp
Itgb2
Mpeg1

Supplemental Figure 1: Alzheimer's disease mouse model DE genes. (A) Number of DE genes between 5xFAD mouse model of Alzheimer's disease (AD) and C57BL/6 J wildtype littermates in young adult (4 months) and aged (18 months), male and female hippocampus, from Forner et al. (Forner et al., 2021). Lowly expressed genes were removed by retaining only genes with counts per million > 0.1 in at least fourteen samples (23,082 transcripts). For DEA, the analysis package edgeR (Robinson et al., 2010) was used, with an FDR < 0.05 cutoff. Chi-square test, young male vs female X^2 = 71.6 and aged male versus female X^2 = 733.4, **** indicates p < 0.00001. (B) Venn diagram showing number of genes we found to be DE with aging in both sexes and the number of genes DE in both sexes in the AD mouse hippocampus from Forner et al., 2021. Box shows the identity of the 13 genes that were DE with aging in our dataset and showed AD-related gene expression changes in young adult and aged hippocampus.

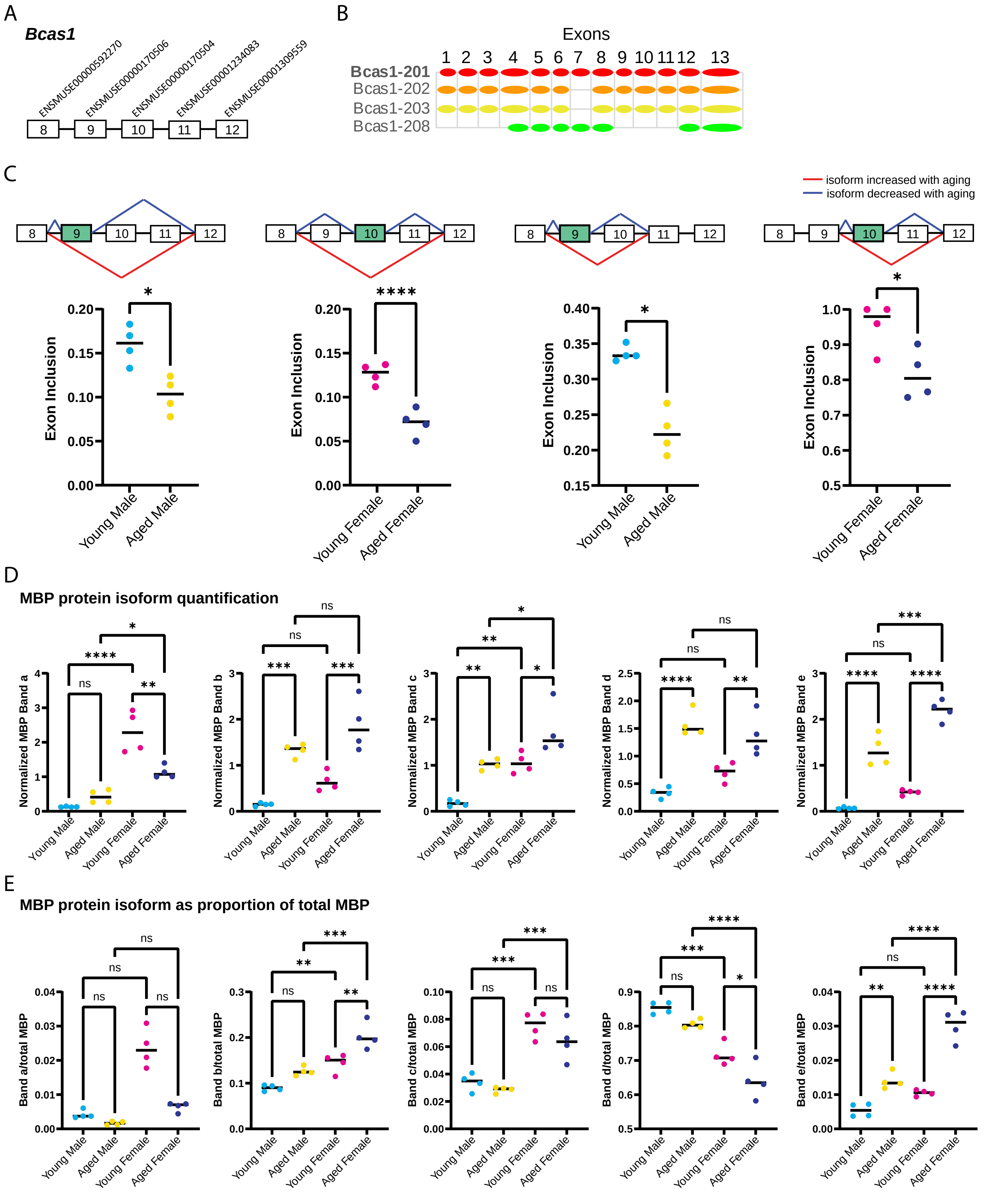
A
Bcas1
ENSMUSE00000592270
ENSMUSE00000170506
ENSMUSE00000170504
ENSMUSE00001234083
ENSMUSE00001309559
B
Exons
Bcas1-201
Bcas1-202
Bcas1-203
Bcas1-208
C
isoform increased with aging
isoform decreased with aging
Exon Inclusion
Young Male
Aged Male
Young Female
Aged Female
D
MBP protein isoform quantification
Normalized MBP Band a
Normalized MBP Band b
Normalized MBP Band c
Normalized MBP Band d
Normalized MBP Band e
E
MBP protein isoform as proportion of total MBP
Band a/total MBP
Band b/total MBP
Band c/total MBP
Band d/total MBP
Band e/total MBP

Supplemental Figure 2: Aging-associated alternative splicing of myelin sheath genes *Bcas1* and *Mbp*. (A) *Bcas1* exons involved in aging-related alternative splicing events in the hippocampus. (B) *Bcas1* protein-coding splice variants from Ensembl (Cunningham et al., 2021) release 102 with a diagram of included exons. Bcas1-201 is bold to indicate Ensembl's designation as a stable, reviewed, and high quality transcript annotation. (C) Diagram and exon inclusion values for each *Bcas1* aging-related alternative splicing event determined by rMATS. In the first two panels, skipping of exons 9-11 is depicted (copy of Fig. 4B *Bcas1* panels). For male and female hippocampus, one additional aging-related alternative splicing event was detected for each (right two panels), in which exons 9 and 10 were skipped with aging. The alternative splicing in all these events skip exon 9 or exon 10, and it is unclear if these events defined by rMATS represent the increasing expressing of isoform Bcas1-208 versus Bcas1-201 with aging in both males and females, or if other unannotated *Bcas1* transcripts are produced. * indicates $p < 0.05$, *** indicates $p < 0.001$. (D) Western blot analysis for MBP protein isoforms in young adult and aged hippocampus from Fig. 4C. MBP band "a" young female vs aged female $p = 0.001$; young male vs young female $p < 0.0001$; aged male vs aged female $p = 0.043$. MBP band "b" young male vs aged male $p < 0.001$; young female vs aged female $p < 0.001$. MBP band "c" young male vs aged male $p = 0.008$; young female vs aged female $p = 0.027$; young male vs young female $p = 0.006$; aged male vs aged female $p = 0.020$. MBP band "d" young male vs aged male $p < 0.0001$; young female vs aged female $p = 0.010$. MBP band "e" young male vs aged male $p < 0.0001$; young female vs aged female $p < 0.0001$; aged male vs aged female $p < 0.001$. ANOVA with Sidak's multiple comparison test was used for all tests. * indicates $p < 0.05$, ** indicates $p < 0.01$, *** indicates $p < 0.001$, **** indicates $p < 0.0001$ and ns indicates not significant. (E) Same as in D, except each band was normalized to total MBP to determine how each isoform's relative proportion changed with aging. MBP band "a" $p > 0.05$. MBP band "b" young female vs aged female $p = 0.003$; young male vs young female $p = 0.006$; aged male vs aged female $p < 0.001$. MBP band "c" young male vs young female $p < 0.001$; aged male vs aged female $p < 0.001$. MBP band "d" young female vs aged female $p = 0.023$; young male vs young female $p < 0.001$; aged male vs aged female $p < 0.0001$. MBP band "e" young male vs aged male $p = 0.003$; young female vs aged female $p < 0.0001$; aged male vs aged female $p < 0.0001$. ANOVA with Sidak's multiple comparison test was used for all tests.

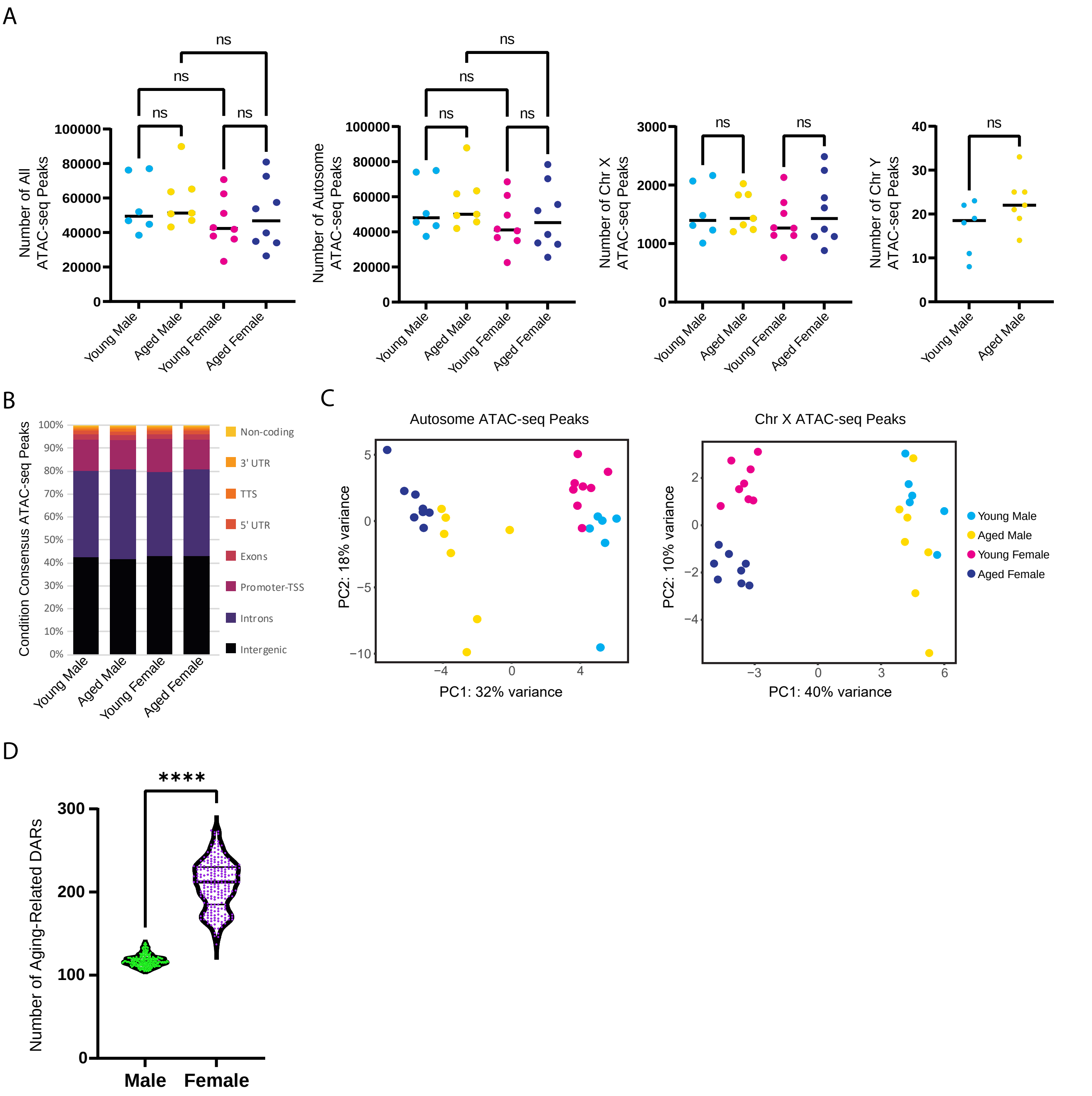
A
ns
Number of All ATAC-seq Peaks
100000
80000
60000
40000
20000
0
Young Male
Aged Male
Young Female
Aged Female
Number of Autosome ATAC-seq Peaks
Number of Chr X ATAC-seq Peaks
3000
2000
1000
Number of Chr Y ATAC-seq Peaks
40
30
20
10
B
Condition Consensus ATAC-seq Peaks
100%
90%
80%
70%
60%
50%
40%
30%
20%
10%
0%
Non-coding
3' UTR
TTS
5' UTR
Exons
Promoter-TSS
Introns
Intergenic
C
Autosome ATAC-seq Peaks
PC2: 18% variance
PC1: 32% variance
Chr X ATAC-seq Peaks
PC2: 10% variance
PC1: 40% variance
Young Male
Aged Male
Young Female
Aged Female
D
****
Number of Aging-Related DARs
300
200
100
0
Male
Female

Supplemental Figure 3: Number and annotation of ATAC-seq peaks are similar across conditions. (A) From left to right, number of ATAC-seq peaks with FDR < 0.05 located on all chromosomes, autosomes, chromosome X or chromosome Y in the hippocampus of 6 young adult male, 7 aged male, 8 young adult female and 8 aged female mice. All peaks: Kruskal-Wallis with Dunn's multiple comparisons test adjusted p > 0.05 for all comparisons. Autosome peaks: one-way ANOVA with Sidak's multiple comparison test adjusted p > 0.05 for all comparisons. Chromosome X peaks: one-way ANOVA with Sidak's multiple comparison test adjusted p > 0.05 for both comparisons. Chromosome Y peaks: unpaired t-test p > 0.05. ns = not significant. (B) Genome annotation of condition consensus ATAC-seq peaks. These peaks were generated from sample peaks sets and merged to generate the total consensus peak set. (C) Principle component analysis of ATAC-seq fragments in the total consensus peaks located on either autosomes (left) or chromosome X (right). There were too few total consensus ATAC-seq peaks located on chromosome Y to perform principal components analysis. (D) Number of significant aging-related differentially accessible regions (DARs) in male and female hippocampus for 224 combinations of samples which included all 6 young male and all 7 aged male samples with different combinations of 6 young female and 7 aged female samples; Mann-Whitney U = 2, **** indicates p < 0.0001.

A

Enriched Motifs in Regions that Opened with Aging in Both Sexes

Motif		Sequences with Motif in Target : Background
HOXA2:	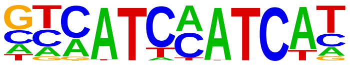	8.4% : 0.8%
DMC1:	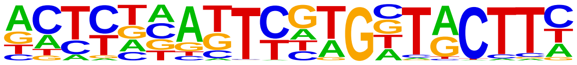	6.0% : 0.5%
MEF2A:	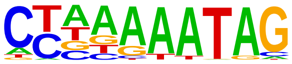	14.5% : 5.6%
MYNN:	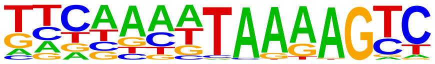	7.2% : 1.7%
MEF2C:	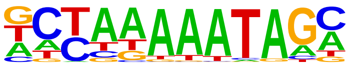	14.5% : 6.0%
HOXA1:	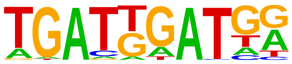	8.4% : 2.4%
ZNF263:	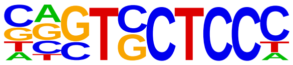	27.7% : 16.0%
ETS1:	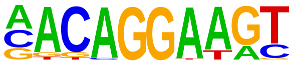	8.4% : 2.6%
OLIG2:	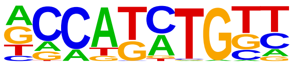	49.4% : 35.6%

B

LINE-1 Regions Opened with Aging in Both Sexes

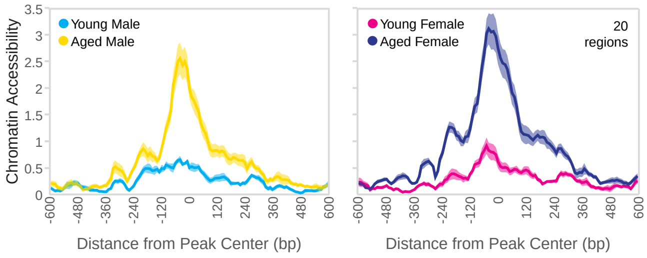

C

Autosome Full-length Intact LINE-1 regions

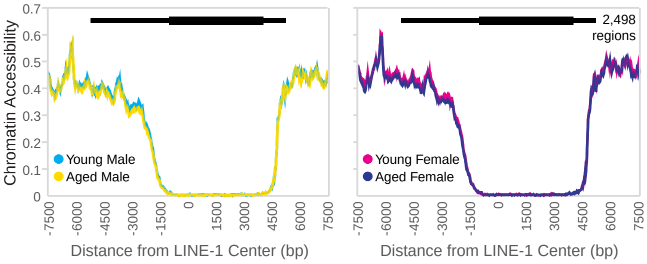

ChrX Full-length Intact LINE-1 regions

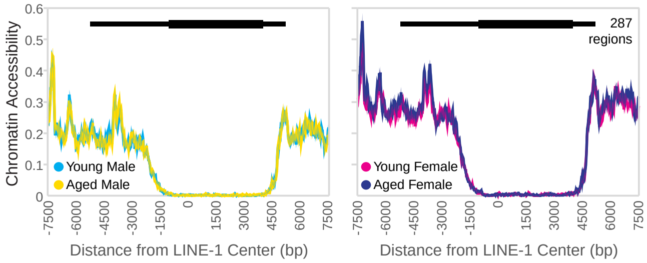

Supplemental Figure 4: Aging-associated changes in chromatin accessibility. (A) Motifs enriched in regions that gained accessibility with aging in both female and male hippocampus (FDR < 0.05). (B) ATAC-seq profiles for regions containing LINE-1 elements that showed increased accessibility with aging in both males and females (FDR < 0.05). Solid lines indicate the average of each condition’s normalized histogram (n = 6 young adult male, 7 aged male, 8 young adult female, 8 aged female) with shading indicating s.e.m. (C) ATAC-seq profiles (as in B) for full-length intact LINE-1 elements on autosomes (above) and on the X chromosome (below). Shown above each ATAC-seq plot is an aligned schematic of a full-length LINE-1 element.

A

Promoter-TSS Motif Enrichment

Female-biased		Male-biased	
**Young**	**Aged**	**Young**	**Aged**
none	none	GFX:<br>ZBTB33:<br>ELK1:<br>NRF1:	SP5:<br>KLF14:<br>KLF1:<br>KLF6:<br>KLF5:<br>MAZ:<br>SP2:<br>KLF3:<br>KLF9:<br>GFX:

B

Non-Promoter Peaks Motif Enrichment

Female-biased		Male-biased	
**Young**	**Aged**	**Young**	**Aged**
FRA2:	CTCF:<br>CTCFL:<br>ATOH1:<br>NEUROD1:<br>NEUROG2:<br>TLX:<br>SMAD3:<br>EN1:<br>AP-1:	none	CTCF:<br>HOXA10:<br>ZNF382:<br>MEF2D:

Supplemental Figure 5: Motifs in regions with sex-bias in chromatin accessibility. (A) Names and motifs enriched in differentially accessible autosome promoter-TSS regions between female and male hippocampus (FDR < 0.05). Zero and three autosome promoter-TSS regions showed a female-bias in accessibility in young adult and aged hippocampus, respectively, yielding no significantly enriched motifs. There were 26 motifs significantly enriched in regions showing male-bias in aged hippocampus, and the top 10 most significant are shown. (B) Name and enriched motifs as in (A), but for non-promoter regions that were differentially accessible between female and male hippocampus. No enriched motifs were found for non-promoter regions that showed a male-bias in accessibility in young animals.

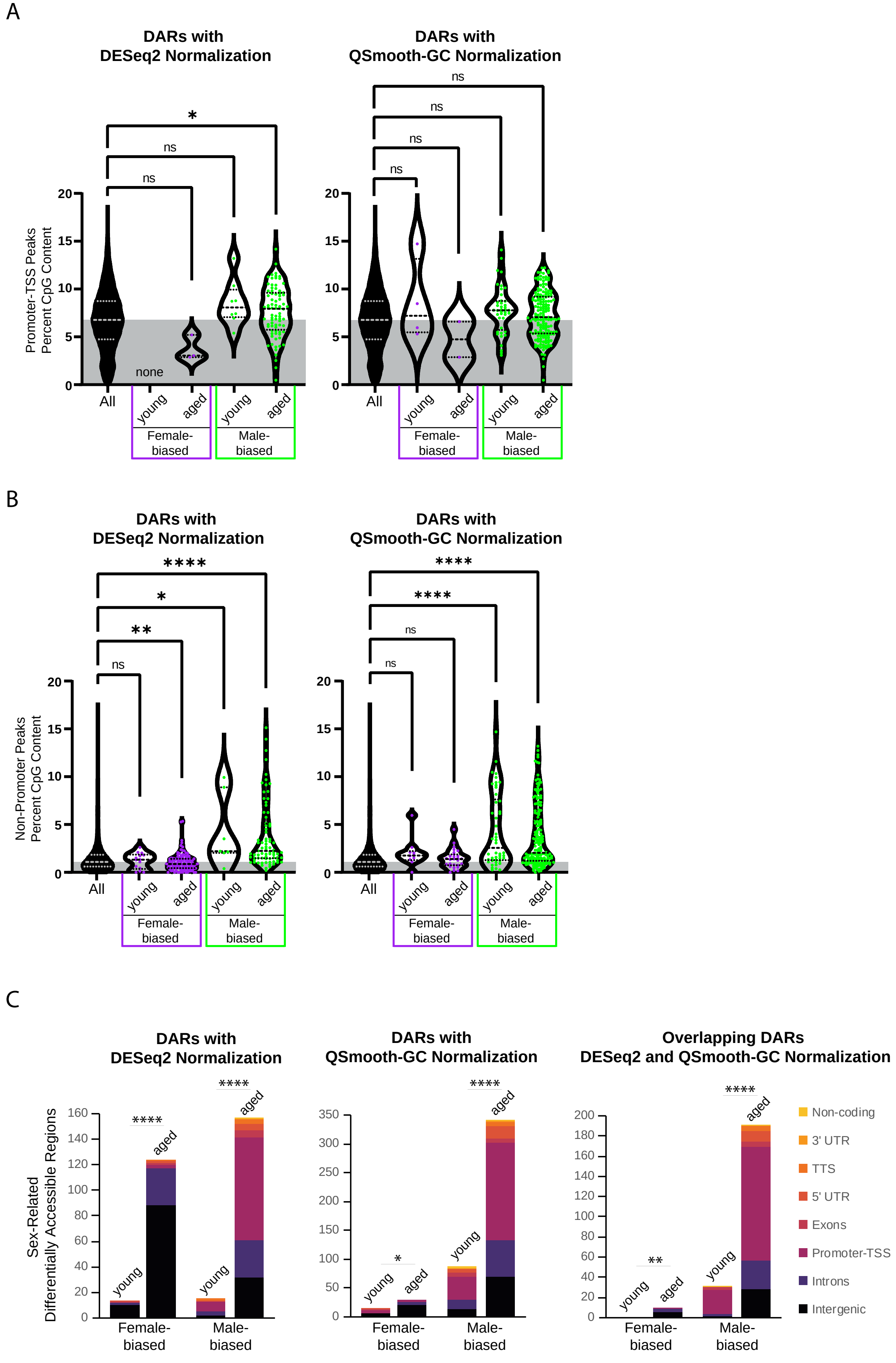
A
DARs with
DESeq2 Normalization
DARs with
QSmooth-GC Normalization
Promoter-TSS Peaks
Percent CpG Content
ns
*
none
All
young
aged
Female-
biased
Male-
biased
B
DARs with
DESeq2 Normalization
DARs with
QSmooth-GC Normalization
Non-Promoter Peaks
Percent CpG Content
****
**
C
DARs with
DESeq2 Normalization
DARs with
QSmooth-GC Normalization
Overlapping DARs
DESeq2 and QSmooth-GC Normalization
Sex-Related
Differentially Accessible Regions
Non-coding
3' UTR
TTS
5' UTR
Exons
Promoter-TSS
Introns
Intergenic

Supplemental Figure 6: Male bias in chromatin accessibility at promoters and CpG-rich regions. (A) Violin plots of ATAC-seq autosome promoter-TSS regions' CpG content. CpG content was calculated using Homer (Heinz et al., 2010) for all ATAC-seq promoter-TSS regions (All; 9,541 regions) and compared to those autosome promoter regions that were differentially accessible with DESeq2 normalization (left panel) or QSmooth-GC normalization (right panel). For data in both panels, Kruskal-Wallis tests with Dunn's multiple comparison tests were used. Average ± s.e.m., All promoter ATAC-seq regions CpG = 6.7% ± 0.0. For autosome promoter DARs with DESeq2 normalization: more open in young adult female (0), more open in aged females (3), more open in young adult males (8) and more open in aged males (80); open aged female CpG = 3.7% ± 0.8, $p = 0.18$; open young male CpG = 8.5% ± 0.8, $p = 0.27$; open aged male CpG = 7.6% ± 0.3, $p = 0.02$. For autosome promoter DARs with QSmooth-GC normalization: more open in young females (4), more open in aged females (2), more open in young males (41) and more open in aged males (167); more open young female CpG = 8.6% ± 2.1, $p = 1$; more open aged female CpG = 4.7% ± 1.9, $p = 1$; more open young male CpG = 7.8% ± 0.4, $p = 0.10$; more open aged male CpG = 7.3% ± 0.2, $p = 0.09$.). (B) CpG content as in (A), but for non-promoter regions, all ATAC-seq non-promoter regions (All; 82,692 regions) CpG = 1.6% ± 0.0 (average ± s.e.m.). For data in both panels, Kruskal-Wallis tests with Dunn's multiple comparison tests were used. For non-promoter autosome DARs with DESeq2 normalization, more open in young females (14), more open in aged females (121), more open in young males (7) and more open in aged males (77); open young female CpG = 1.2% ± 0.2, $p = 1$; open aged female CpG = 1.0% ± 0.1, $p = 0.002$; open young male CpG = 4.2% ± 1.4, $p = 0.04$; open aged male CpG = 3.8% ± 0.4, $p < 0.0001$. For non-promoter DARs with QSmooth-GC normalization: more open in young females (10), more open in aged females (27), more open in young males (46) and more open in aged males (174); more open young female CpG = 2.0% ± 0.5, $p = 0.28$; more open aged female CpG = 1.4% ± 0.2, $p = 1$; more open young male CpG = 4.4% ± 0.6, $p < 0.0001$; more open aged male CpG = 3.7% ± 0.2, $p < 0.0001$. (C) Genome annotation of autosome differently accessible regions (DARs) between female and male hippocampus. Left panel shows annotation of regions found to be differentially accessible using DESeq2 normalization (copy of Fig. 8D). Chi-square test, young vs aged $X^2 = 87.7$ more open female, $X^2 = 117.3$ more open male, $p < 0.00001$. Middle panel shows annotation of regions found to be differentially accessible using QSmooth-GC Normalization (FDR < 0.05). Chi-square test, young vs aged $X^2 = 5.2$ more open female, $p = 0.021$; $X^2 = 151.1$ more open male, $p < 0.0001$. Right panel shows annotation of regions found to be differentially accessible using either DESeq2 or QSmooth-GC Normalization (FDR < 0.05). Chi-square test, young vs aged $X^2 = 7.4$ more open female, $p < 0.01$, $X^2 = 113.5$ more open male, $p < 0.00001$. * indicates $p < 0.05$, ** indicates $p < 0.01$, **** indicates $p < 0.0001$.

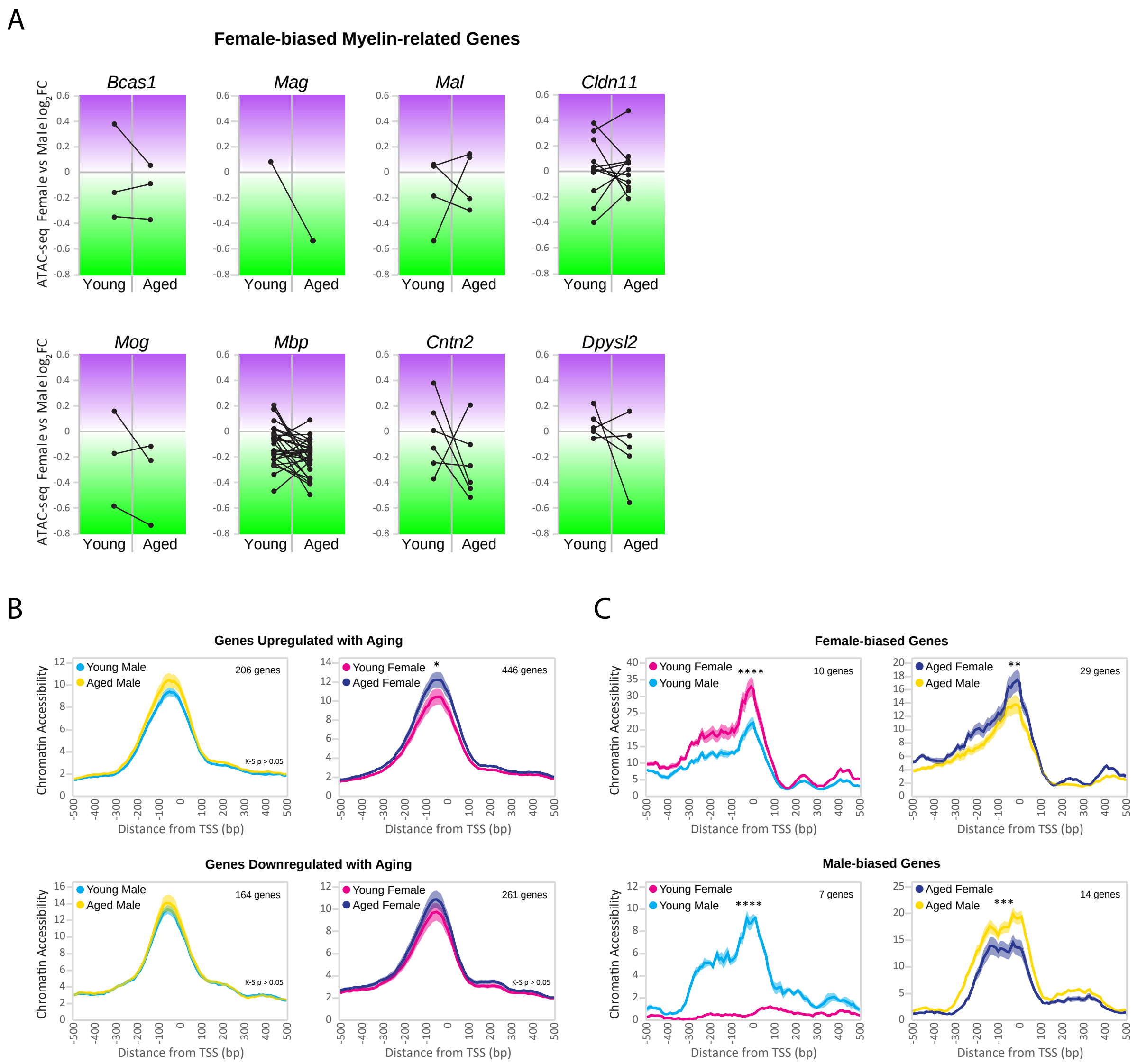
A
Female-biased Myelin-related Genes
Bcas1
Mag
Mal
Cldn11
Mog
Mbp
Cntn2
Dpysl2
ATAC-seq Female vs Male log2FC
Young
Aged
B
Genes Upregulated with Aging
Genes Downregulated with Aging
Young Male
Aged Male
Young Female
Aged Female
206 genes
446 genes
164 genes
261 genes
K-S p > 0.05
Chromatin Accessibility
Distance from TSS (bp)
C
Female-biased Genes
Male-biased Genes
10 genes
29 genes
7 genes
14 genes

Supplemental Figure 7: Chromatin accessibility near myelin-related genes and correlation of DE with ATAC-seq signal at gene TSSs. (A) Sex-biased chromatin accessibility in young and aged hippocampus for ATAC-seq regions annotated to female-biased myelin-related genes. Shown are the ATAC-seq female vs male $\log_2$FC values for each associated ATAC-region, with positive values indicating female-biased accessibility and negative values indicating male-biased accessibility. None of these regions showed sex-biased accessibility in young or aged hippocampus. (B) ATAC-seq profiles surrounding the TSS of genes that were upregulated with aging (top row) or downregulated with aging (bottom row). Solid lines indicate the average of each condition's normalized histogram (n = 6 young adult male, 7 aged male, 8 young adult female, 8 aged female) with shading indicating s.e.m. (upregulated with aging in male: Kolmogorov-Smirnov D = 0.17, p = 0.11; female: Kolmogorov-Smirnov D = 0.21, p = 0.03; downregulated with aging in male: Kolmogorov-Smirnov D = 0.11, p = 0.59; female: Kolmogorov-Smirnov D = 0.18, p = 0.08). (C) ATAC-seq profiles surrounding the TSS of genes that were higher expressed in female hippocampus (top row) or higher expressed in male hippocampus (bottom row). Solid lines indicate the average of each condition's normalized histogram (n = 6 young male, 7 aged male, 8 young female, 8 aged female) with shading indicating s.e.m. (higher expressed in female young: Kolmogorov-Smirnov D = 0.26, p = 0.003; aged: Kolmogorov-Smirnov D = 0.22, p < 0.0001; higher expressed in male young: Kolmogorov-Smirnov D = 0.82, p < 0.0001; aged: Kolmogorov-Smirnov D = 0.31, p = 0.0001).* indicates p < 0.05, ** indicates p < 0.01, *** indicates p < 0.001, **** indicates p < 0.0001.

Supplemental Table 1: RNA-seq DE data. RNA-seq gene data from young adult and aged, female and male hippocampus.

Supplemental Table 2: Alternative splicing data. Alternative splicing events from young adult and aged, female and male hippocampus. SE = skipped exon, A5SS = alternative 5' splice site, A3SS = alternative 3'splice site, MXE = mutually exclusive exons, RI = retained intron.

Supplemental Table 3: ATAC-seq data. ATAC-seq total consensus peaks data from young adult and aged, female and male hippocampus.

References

Cunningham, F., Allen, J.E., Allen, J., Alvarez-Jarreta, J., Amode, M R., Armean, Irina M., Austine-Orimoloye, O., Azov, Andrey G., Barnes, I., Bennett, R., *et al.* (2021). Ensembl 2022. Nucleic Acids Research *50*, D988-D995.
Forner, S., Kawauchi, S., Balderrama-Gutierrez, G., Kramár, E.A., Matheos, D.P., Phan, J., Javonillo, D.I., Tran, K.M., Hingco, E., da Cunha, C., *et al.* (2021). Systematic phenotyping and characterization of the 5xFAD mouse model of Alzheimer's disease. Scientific Data *8*, 270.
Heinz, S., Benner, C., Spann, N., Bertolino, E., Lin, Y.C., Laslo, P., Cheng, J.X., Murre, C., Singh, H., and Glass, C.K. (2010). Simple combinations of lineage-determining transcription factors prime cis-regulatory elements required for macrophage and B cell identities. Mol Cell *38*, 576-589.
